# Transcriptomic and pathological analysis of the hnRNP network reveals glial involvement in frontotemporal lobar degeneration pathological subtypes

**DOI:** 10.1093/braincomms/fcag197

**Published:** 2026-06-01

**Authors:** Ariana Gatt, Yazead Buhidma, Katherine Fodder, Jack Humphrey, Sandrine C Foti, Barbara Frias, Bridget C Benson, Priya Gami-Patel, Lauren M Gittings, Christina E Toomey, Tammaryn Lashley

**Affiliations:** Department of Neurodegenerative Diseases, UCL Queen Square Institute of Neurology, University College London, London WC1N 1PJ, UK; Department of Neurodegenerative Diseases, UCL Queen Square Institute of Neurology, University College London, London WC1N 1PJ, UK; Department of Neurodegenerative Diseases, UCL Queen Square Institute of Neurology, University College London, London WC1N 1PJ, UK; Nash Family Department of Neuroscience and Friedman Brain Institute, Icahn School of Medicine at Mount Sinai, NewYork City, NY 10029, USA; Department of Neurodegenerative Diseases, UCL Queen Square Institute of Neurology, University College London, London WC1N 1PJ, UK; Department of Neurodegenerative Diseases, UCL Queen Square Institute of Neurology, University College London, London WC1N 1PJ, UK; Department of Neuroscience, Sheffield Institute for Translational Neuroscience (SITraN), University of Sheffield, Sheffield S10 2HQ, UK; Department of Pathology, Amsterdam Neuroscience, Amsterdam University Medical Centers, Amsterdam, The Netherlands; Department of Translational Neuroscience, Barrow Neurological Institute, Phoenix AZ 85013, USA; Department of Neurodegenerative Diseases, UCL Queen Square Institute of Neurology, University College London, London WC1N 1PJ, UK; Department of Neurodegenerative Diseases, UCL Queen Square Institute of Neurology, University College London, London WC1N 1PJ, UK

**Keywords:** frontotemporal lobar degeneration, RNA-binding proteins, dementia, astrocytes, oligodendrocytes

## Abstract

Frontotemporal dementia is a neurodegenerative disorder with a strong heritable component. Frontotemporal lobar degeneration refers to the pathological changes seen in frontotemporal dementia, characterized by atrophy of the frontal and temporal lobes and the presence of abnormal protein inclusions. In the case of frontotemporal lobar degeneration with hyperphosphorylated TDP-43 positive inclusions (FTLD-TDP), five pathological subtypes (A, B, C, D and E) are observed based on the types and distribution of inclusions found in the brain. In all subtypes, there tends to be a large variability in the number of pathological inclusions observed between cases, with limited correlation to clinical manifestations.

TDP-43 is an RNA-binding protein belonging to the heterogeneous nuclear ribonucleoprotein (hnRNP) family, which along with other hnRNPs, modulates multiple aspects of RNA processing. HnRNPs other than TDP-43 have been implicated in several neurological diseases, including Amyotrophic Lateral Sclerosis, FTLD-TDP, frontotemporal lobar degeneration with fused in sarcoma (FTLD-FUS) and Alzheimer’s disease. Multiple hnRNPs have been found in pathological inclusions in specific subtypes of FTLD-TDP, suggesting potential roles in the disease process. The role of the hnRNP network in frontotemporal lobar degeneration disease pathogenesis, however, has not yet been investigated. This study aimed to comprehensively evaluate the presence and expression of hnRNP proteins in two pathological subtypes of sporadic FTLD-TDP (A and C) as well as the genetic form FTLD-TDP A *C9orf72* using immunohistochemistry and gene expression analysis by single-nuclei RNA-sequencing.

We found that there was great variability in the frequency of TDP-43 pathology across and within FTLD-TDP pathological subtypes. Our findings suggest that distinct global transcriptomic profiles may underlie the different pathological subtypes of FTLD-TDP. The most prominent transcriptomic changes were observed in oligodendrocytes and astrocytes, involving multiple hnRNPs across frontotemporal lobar degeneration subtypes compared to controls. Transcriptomic co-expression analysis further revealed that glial clusters were more strongly associated with RNA-processing dysfunction and contributed to disease classification. Together, these findings highlight the involvement of the hnRNP network and glial-specific RNA-processing alterations in FTLD-TDP pathophysiology, offering new insight into the molecular distinctions between pathological subtypes and potential targets for future investigation.

## Teaser

Gatt *et al.* report that frontotemporal lobar degeneration with TDP-43 (FTLD-TDP) inclusions showed variable pathology burden across subtypes. They analysed hnRNP expression in FTLD-TDP A, C and *C9orf72* cases using immunohistochemistry and single-nuclei RNA sequencing. Distinct glial transcriptomic profiles and RNA-processing disruptions emerged, highlighting subtype-specific molecular differences underlying disease pathogenesis.

## Graphical Abstract

**Graphical Abstract:**
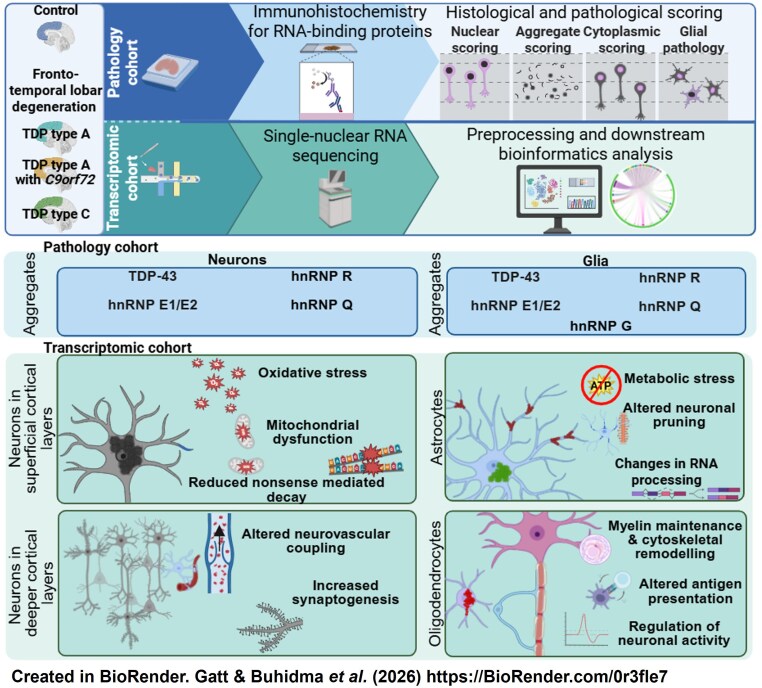

## Introduction

Frontotemporal dementia (FTD) comprises three main clinical syndromes: behavioural variant FTD (bvFTD), semantic dementia and progressive non-fluent aphasia, and can overlap with motor neuron disease/amyotrophic lateral sclerosis (MND/ALS) (FTD-MND), corticobasal syndrome and progressive supranuclear palsy.^1^ Around 30–50% of cases are familial, most often caused by mutations in *MAPT*, *GRN*, *C9orf72* and *TBK1*.^2-4^

Frontotemporal lobar degeneration (FTLD) is the pathological umbrella term for these disorders and is defined by tau-, TDP-43- or FUS-positive inclusions.^5^ Following the study by Neumann *et al*.^6,^ which identified hyperphosphorylated TDP-43 inclusions, FTLD-TDP has been subdivided into five types (A–E), each characterized by distinct combinations of neuronal intranuclear inclusions (NIIs), neuronal cytoplasmic inclusions (NCIs), dystrophic neurites (DNs), oligodendroglial inclusions and granulofilamentous neuronal inclusions.^7,8^ Type A features abundant neuronal cytoplasmic inclusions and dystrophic neurites with variable neuronal intranuclear inclusions in layer II; Type B has numerous neuronal cytoplasmic inclusions across layers; Types A and B are associated with *C9orf72* mutations. Type C shows corkscrew-like dystrophic neurites across cortical layers and has no known genetic link. Type D, associated with mutations in the Valosin-Containing Protein (*VCP*) gene, contains neuronal intranuclear inclusions, dystrophic neurites and occasional neuronal cytoplasmic inclusions. Type E presents granulofilamentous neuronal inclusions, grains and oligodendroglial inclusions.

TDP-43 is a heterogeneous nuclear ribonucleoprotein (hnRNP) that interacts with other hnRNPs through its C-terminal tail.^9-11^ Around 20 major hnRNPs (A1-U) share overlapping domains and functions in nucleic acid metabolism, including transcription, RNA processing, nuclear export, localization, translation and mRNA stability.^12-16^ Dysregulated hnRNP activity has been implicated in ALS, FTLD-TDP and Alzheimer’s disease.^17-20^ Several hnRNPs have been linked to FTLD pathology: hnRNP E2 accumulates with TDP-43 in dystrophic neurites and inclusions in FTLD-TDP types A and C,^21,22^ hnRNP A3 localizes to dipeptide-repeat-containing inclusions in *C9orf72* cases^23,24^ and hnRNP A3, H1 and H3 interact with hexanucleotide repeat expansions in models.^25^ HnRNP K also mislocalizes to the cytoplasm in neurons lacking TDP-43 pathology across neurodegenerative diseases.^25,26^ Despite growing evidence of their involvement, these hnRNPs remain understudied relative to TDP-43 in FTLD.

This study uses immunohistochemistry and single-nucleus RNA sequencing (snRNA-seq) to profile hnRNP expression and localization in three FTLD-TDP subtypes (sporadic TDP-A, *C9orf72*-associated TDP-A and TDP-C). We aimed to (1) define the diversity of TDP-43 pathology within subtypes, (2) assess hnRNP aggregation and mislocalization, (3) characterize transcriptomic changes across cell types and (4) evaluate concordance between transcript and protein alterations. Our findings show limited correspondence between cortical and hippocampal pTDP-43 pathology and suggest subtype-specific differences in hippocampal burden. Several hnRNPs, including hnRNP G and Q, exhibit cytoplasmic mislocalization and inclusions, with hnRNP Q also accumulating in microglia in TDP-C. SnRNA-seq and co-expression analyses identify glial-enriched modules, particularly astrocytic and oligodendrocytic, associated with diagnosis and aggregate burden, highlighting RNA-splicing dysfunction as a shared pathway. Overall, these results emphasize an important glial contribution to hnRNP-related mechanisms in FTLD.

## Materials and methods

### Cases

Brains were donated to Queen Square Brain Bank for Neurological Disorders, UCL Queen Square Institute of Neurology (QSBB). Cases used included FTLD-TDP type A (17 cases: TDP A 8 cases, TDP A-C9 9 cases) and TDP C (11 cases), which were diagnosed using the pathological criteria outlined by Mackenzie *et al*.^27^ This includes phosphorylated TDP-43 (pTDP43) positive inclusions characterized by their morphology and localization across brain regions. Our study also included neurologically normal controls which were devoid of TDP-43 pathology and had no neurological clinical manifestations (14 cases). Ethical approval for the study was obtained from the National Hospital for Neurology and Neurosurgery Local Research Ethics Committee. A demographic summary of all cases used in this study is shown in Table 1 and extended case demographics in Supplementary Table 1. Supplementary Table 1 also highlights which cases were used in the individual analyses.

**Table 1:** A summary table of the case demographics for cases used in the study

Disease group	No. of cases	M/F	Age at onset (y ± SD)	Age at death (y ± SD)	Disease Duration (y ± SD)	PMI (hrs ± SD)
TDP A	8	5/3	65.5 ± 12.7	72 ± 12.1	6.5 ± 3.3	43.6 ± 29.9
TDP A-*C9*	9	5/4	57.2 ± 7.16	64 ± 8.37	6.8 ± 2.8	69.3 ± 28:7
TDP C	11	7/4	60.3 ± 8.6	71.7 ± 5.3	11.5 ± 6	45.8 ± 22
Controls	14	5/9	n/a	79.3 ± 15.7	n/a	80.9 ± 37.4

Summarized are the number of cases per pathological subtype, male to female ratio (M/F), age at onset, age at death and disease duration in years ± standard deviation (SD). PMI is summarized as years ± standard deviation (SD).

### Single-nucleus RNA-seq data and analysis

The cases used in this analysis are highlighted in Supplementary Table 1. Frozen frontal grey matter was transferred directly onto ice-cold sucrose buffer and homogenized by hand. The homogenate was layered on the top of a sucrose cushion, and nuclei were recovered after centrifugation. Nuclei were resuspended in PBS containing BSA (to prevent clumping) and RNase inhibitors, counted and diluted to 1000 nuclei/μl. Sequencing libraries were prepared using the ‘single-cell 3’ library kit v3’ with the Chromium instrument (10X Genomics). Aiming to obtain libraries for ∼5000 independent nuclei per sample, libraries were sequenced on an Illumina NovaSeq6000 instrument (UCL Genomics), targeting an average depth of at least 100 000 reads/nucleus.

Raw sequencing data were aligned to the human reference genome (hg38) and processed through CellRanger (10X Genomics) without the inclusion of intronic reads. To assign each transcript read to its respective nucleus, we performed alignment to the transcriptome, counted reads and processed the unique molecular identifier (UMI) tag to reduce PCR bias. The resulting gene count table was analysed using the Seurat package (v.5).^28^ Quality control was performed to remove low-quality cells based on the number of detected genes and UMIs: nuclei with fewer than 200 or more than 2500 detected genes, or with more than 5% of reads mapping to mitochondrial genes, were excluded. Cells with unusually high UMI counts, suggesting potential doublet events, were also excluded using scDblFinder.^29^ Using SCTransform,^30^ gene expression data were normalized to adjust for differences in sequencing depth between cells and scaled to correct for cell-to-cell variation in total UMI count. Mitochondrial gene content was regressed out to minimize confounding sources of variation. Batch effects were corrected using the Harmony algorithm to ensure consistency across sequencing batches. Principal component analysis (PCA) was performed on the scaled data to reduce the dimensionality of the dataset. Based on analysis of an elbow plot, the top 20 principal components were selected for downstream analyses. The PCA-generated principal components were used for clustering cells using the graph-based Louvain algorithm implemented in Seurat. Cluster annotation was performed using canonical marker genes for neural cell types sourced from brain-focused transcriptomic databases and relevant literature.^31,32^ Cell types were annotated by comparing the expression pattern of marker genes within each cluster.

The snRNA-seq data were visualized using Uniform Manifold Approximation and Projection (UMAP) to view the nuclei clusters in two dimensions. Clusters were separated into the main neural cell types and differential gene expression analysis was performed to identify genes significantly differentially expressed between identified cell clusters of each disease subtype against controls using the ‘FindMarkers’ function in Seurat with Wilcoxon rank-sum analysis, with statistical significance being defined as a false discovery rate (FDR) of < 0.05 and minimum cut-off of three nuclei per group.

### IPA pathway analysis

Significant differentially expressed genes (DEGs) were input into Qiagen Ingenuity Pathway Analysis software (IPA), mapped to corresponding human gene symbols from GenBank and other human gene databases (HUGO, HGNC and Entrez gene). We then performed Core Analyses on each FTLD subtype (compared to control) across all nuclei clusters. Analyses were conducted using the human IPA Knowledge Base (Genes only) as the reference set, and all supported molecule types were included. IPA’s Upstream Regulator Analysis module predicted transcriptional regulators and signalling molecules influencing gene expression changes. Enrichment of canonical pathways and upstream regulators was assessed using a right-tailed Fisher’s exact test, with significance defined as FDR < 0.05. Activation state predictions were based on IPA’s activation *z*-score algorithm with *z*-score > ± 2 considered sufficiently dysregulated.

### hnRNP target Jaccard similarity analysis

To understand the impact of TDP-43 pathology on superficial cortical neurons, we overlaid TDP-43 targets from the POSTAR3 human database: a cross-linking immunoprecipitation sequencing database that lists proteins that preferentially bind to RNA-binding proteins (RBPs). The Jaccard similarity index assessed overlap in hnRNP gene targets across DEGs in each cell cluster, with targets retrieved from the database.^33^ This allowed evaluation of shared regulatory mechanisms across clusters and which neural cells exhibited the most overlap.

### hdWGCNA analysis

High-dimensional weighted gene co-expression network analysis (hdWGCNA) was performed to assess gene co-expression modules in our dataset.^34^ Data were first aggregated into metacells to enhance data quality, reduce sparsity and improve robustness of network analysis. Data were log-normalized and scaled to control for differences in sequencing depth and technical variance. Gene co-expression modules were identified with a soft-thresholding power selected based on scale-free topology fit indices. A power of 6 was chosen as it was the lowest value at which the scale-free topology model fit (*R*^2^) exceeded 0.8. Subsequently, cell-type enrichment of each module was assessed by testing for over-representation of module genes among cell-type marker genes. Marker genes were identified for each annotated cell type using Seurat’s FindAllMarkers (positive markers only; log_2_ fold change threshold = 0.25), restricted to the hdWGCNA gene universe, and the top 400 markers per cell type (ranked by average log_2_ fold change) were retained. Enrichment for each module-cell type pair was calculated using a hypergeometric test (phyper, testing for overlap greater than expected by chance), with Benjamini–Hochberg correction. Effect sizes were summarized as fold enrichment and significant enrichments were defined as FDR <0.05 with ≥5 overlapping genes. Analysis of relative module eigengene expression across subtypes was performed using the hdWGNCA, ‘FindAllDMEs’ function, where modules were compared to control using a Wilcoxon test. These analyses allowed for the identification of disrupted gene networks associated with specific subtypes. Module-trait correlations were conducted to investigate associations between identified gene modules and clinical and pathological traits. Module eigengenes (MEs) were computed as representative summaries of module expression, and Pearson correlation coefficients were calculated to quantify the strength of association between module eigengenes and traits of interest. Significant correlations were evaluated using adjusted *P*-values derived from two-tailed *t*-tests that were subjected to a Bonferroni adjustment to account for multiple hypothesis testing, thereby identifying biologically relevant modules linked to specific clinical or pathological features.

Modules from hdWGCNA showing significant associations with disease status and pathological burden were analysed for gene ontology (GO) enrichment using ClusterProfiler.^35^ GO terms relating to biological processes, molecular functions and cellular components were ranked by adjusted *P*-values (FDR < 0.05), and enrichment was visualized through dot plots.

To further explore the regulatory architecture of disease-associated modules, hub gene analysis was performed within each module using hdWGCNA. Intramodular connectivity (kME) was calculated as the Pearson correlation between individual gene expression profiles and the module eigengene, representing the module’s overall expression pattern. Genes were ordered by kME values, and from the top 1% were considered putative hub genes due to their strong co-expression with the core module signature. The hub genes associated with RBP mechanisms such as RNA processes, splicing and binding were highlighted for each module network based on the overlap of related GO terms. This approach enabled the identification of central regulators potentially contributing to disease pathogenesis, particularly within astrocytic and oligodendrocyte-enriched modules that showed strong associations with disease classification and pathology scores.

### Immunohistochemistry for hnRNPs

8 *μ*m-thick formalin-fixed paraffin-embedded sections were cut from TDP A cases, TDP A-*C9* (*n* = 7), TDP C (*n* = 8) and neurologically normal control cases (*n* = 6) (Supplementary Table 1). The sections were deparaffinized in xylene and rehydrated using graded alcohols. Endogenous peroxidase activity was blocked using 0.3% H_2_O_2_ in methanol for 10 min followed by pressure cooker pretreatment for 10 min in citrate buffer, pH 6.0. Non-specific binding was blocked using 10% dried milk/Tris buffered saline-Tween before incubating with a hnRNP-derived primary antibody overnight at 4°C. Supplementary Table 2 lists all the antibodies used in this study with their supplier and concentration used. A biotinylated anti-rabbit/mouse IgG antibody (1:200, 30 min, DAKO) was incubated with the sections at room temperature, followed by avidin-biotin complex (30 min, Vector Laboratories). The colour was developed with di-aminobenzidine activated with H_2_O_2_.^36^ Images were taken with a Nikon H550L light microscope. Standard immunohistochemical staining for Aβ and tau were carried out to determine the presence of concomitant pathologies as described previously,^37^ and standard diagnostic criteria for the neuropathological diagnosis of Alzheimer’s disease and the presence of cerebral amyloid angiopathy were used in all cases.^38-42^

### Quantification of phosphorylated TDP-43 pathology

TDP-43 pathology was quantified in the frontal and temporal grey matter and the hippocampal granule cell layer (GCL) using a phospho-TDP-43 (pTDP-43) antibody to selectively label pathological inclusions (Supplementary Table 2). Since pTDP-43 presents as distinct features across FTLD subtypes, frontal and temporal cortex staining was manually quantified by separately counting intranuclear/cytoplasmic inclusions and neurites. The grid feature on QuPath software was used as a visual aid in order to ensure quantification along all six cortical layers of the grey matter per section.^43^ Counts within each grid square were averaged to obtain a pathology measure per case. In the hippocampal granule cell layer, pTDP-43-positive inclusions were counted at 10× magnification and expressed as a percentage of total cells. All scoring was performed by a single blinded observer (AG for cortex; TL for hippocampus).

### Quantification of hnRNP staining

Immunohistochemically stained sections were examined to assess hnRNP localization in the frontal cortex grey matter, selected because it is affected across all FTLD-TDP subtypes and used for gene expression analysis. Neuronal nuclear and cytoplasmic hnRNP staining was scored semi-quantitatively as previously described.^22,24^ All grey matter in each section was evaluated using the following scale: 0 = no staining; 1 = 1–5 cells with weak staining; 2 = 5–10 cells with moderate staining; 3 = >10 cells with strong staining. Pathological inclusions (neuronal cytoplasmic, neuronal intranuclear and dystrophic neurites) were graded as: 0 = none; 0.5 (rare) = 1–5 inclusions per section; 1 (few) = 1–5 per field; 2 (mild) = 5–10 per field; 3 (moderate) = 10–50 per field. Any deviations from normal hnRNP patterns were noted. All scoring was performed by a single blinded observer (TL).

### Statistical analysis

Group comparisons of age at onset, age at death (AAD), *post-mortem* interval (PMI) and duration of illness were made using the Kruskal–Wallis test with *post-hoc* Dunn’s multiple comparison test. pTDP-43 immunostaining data were analysed using GraphPad Prism software (version 10.0). The 28 FTLD patients were stratified according to genetic and pathological subtype for statistical analysis of the effect of each mutation and underlying pathology on the degree and pattern of pTDP-43 staining. Comparisons of semi-quantitative scores for the frequency of pTDP-43-positive inclusions and neurites in neurons of the frontal and temporal cortex, and dentate gyrus (DG) of the hippocampus, with respect to pathological type or genetic mutation, were performed using the Kruskal–Wallis test with *post-hoc* Dunn’s multiple comparison test.

Comparison of rating scores for the inclusion/cytoplasmic/nuclear (cellular localization) immunostaining of the various hnRNPs in the three different pathological subtypes was performed using a Kruskal–Wallis H test (K independent samples) on SPSS software (v.29), a non-parametric method appropriate for comparing ordinal data between more than two independent groups. Specifically, separate Kruskal–Wallis tests were performed to assess differences in protein scores (scored 0–3) across the four disease conditions (Control, TDP A, TDP A-*C9*, TDP C) within each cellular compartment (nucleus, cytoplasm, inclusion). The hnRNP cellular localization was deemed the test variable, whilst the pathological subtype was the grouping variable. The *H* statistic for this test reflects the degree to which the mean ranks differ among groups; a higher *H* value indicates greater differences between groups. In all instances, significance levels for the *P*-value were set at *P* < 0.05.

## Results

### Demographic comparisons

The demographic data pertaining to the cases included in this study are listed in Table 1. There was a statistical difference in *post-mortem* interval between the FTLD subtypes and control groups (*H* = 9.979, *P* = 0.019), though pairwise comparisons revealed no statistically significant difference. There was no statistical difference between age of onset (*H* = 2.505, *P* = 0.286) and disease duration (*H* = 4.606, *P* = 0.099) in the FTLD subtype groups (Supplementary Fig. 1). The mean age at death (AAD) was statistically different between the groups (*H* = 9.632, *P* = 0.022) with the TDP A-*C9* group having a statistically lower mean AAD than controls (Dunn’s multiple comparison, adjusted *P* = 0.012; Supplementary Fig. 1).

### Variation in pTDP-43 pathology burden across and within FTLD pathological subtypes

**Figure 1:**
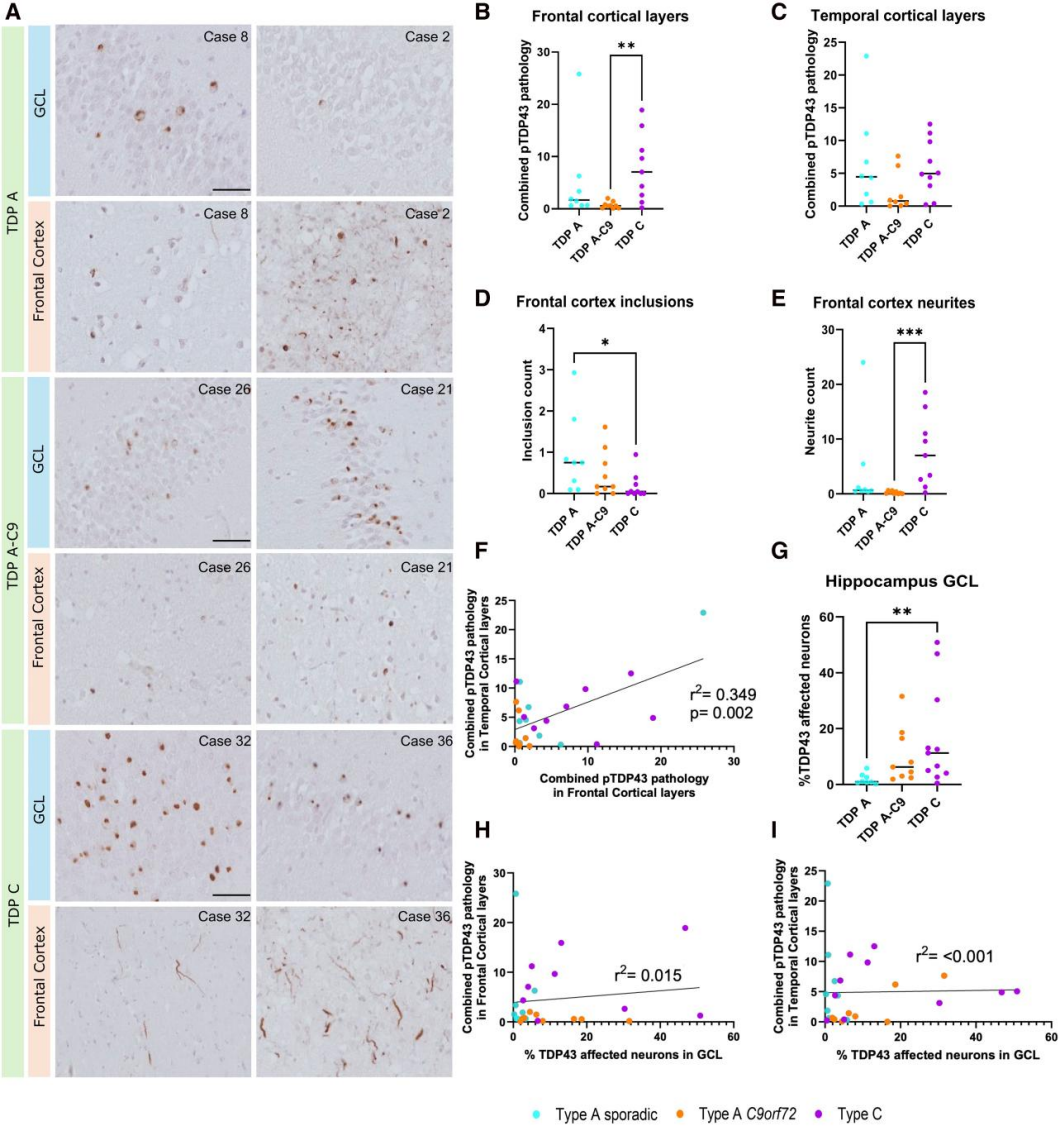
**Quantification of pTDP43 pathology in FTLD-TDP subtypes.** (**A**) Immunohistochemical images showing that the frequency of pTDP-43 pathological inclusions and/or neurites varies greatly between cases and across subtypes. (**B**) Quantification of combined pTDP43 pathology (inclusions and neurites) in the frontal cortical layers across subtypes TDP A (*n* = 8), TDP A-*C9* (*n* = 9) and TDP C (*n* = 11). (**C**) Quantification of combined pTDP43 pathology (inclusions and neurites) in the temporal cortical layers across subtypes TDP A, TDP A-*C9* and TDP C. (**D**) Quantification of pTDP43 positive inclusions in the frontal cortical layers across subtypes TDP A, TDP A-*C9* and TDP C. (**E**) Quantification of pTDP43 positive neurites in the frontal cortical layers across subtypes TDP A, TDP A-*C9* and TDP C. (**F**) Correlation of combined pTDP43 pathology along temporal and frontal cortical layers (simple linear regression, *R*^2^ = 0.349, *P* = 0.002). (**G**) Quantification of pTDP43 pathology in the hippocampal GCL. Values are shown as the percentage of cells affected by pTDP43 inclusions in relation to the total number of cells. (**H**) Correlation of combined pTDP43 pathology along GCL in hippocampus and frontal cortical layers (simple linear regression, *R*^2^ = 0.015, *P* = n.s.). (**I**) Correlation of combined pTDP43 pathology along GCL in hippocampus and temporal cortical layers (simple linear regression, *R*^2^ = <0.001, *P* = n.s.). All comparisons of pathology between FTLD subtypes were analysed by Kruskal–Wallis tests with Dunn’s multiple comparison tests. In relation to all statistical results, * = *P* = 0.01–0.05, ** = *P* = 0.001–0.01 and *** = *P* = <0.001. A *P*-value > 0.05 is deemed n.s. Demographic data of the cases outlined in this figure are listed in Supplementary Table 1. In A–I, each data point represents an individual case. Scale bar represents 50 µm. n.s.—non-significant.

Cases from FTLD-TDP Type A (sporadic and genetic) and Type C showed characteristic pTDP-43 pathology; NCIs, DNs, NIIs (Type A) and long thick DNs (Type C; Fig. 1). Pathology severity varied by case (Supplementary Table 1), so we manually quantified neurites and inclusions to generate semi-quantitative burden scores. Combined pathology was the highest in TDP C within the frontal cortex (Kruskal–Wallis *H* = 10.39, *P* = 0.006, Dunn’s multiple comparison test; Fig. 1A and B), while TDP A-*C9* showed the lowest burden, largely due to absent neurites (Fig. 1B and C). TDP A sporadic cases exhibited the most inclusions (Kruskal–Wallis *H* = 6.142, *P* = 0.046272, Dunn’s multiple comparison test; Fig. 1D), whereas TDP C showed few inclusions but abundant neurites (Kruskal–Wallis *H* = 13.33, *P* = 0.001, Dunn’s multiple comparison test; Fig. 1D and E). Overall, pTDP-43 pathology was greater in frontal than temporal cortex, with moderate correlation observed between regions (*R*^2^ = 0.349, *P* = 0.002; Fig. 1F).

We also quantified pathology in the dentate gyrus GCL, where pTDP-43 inclusions are common. TDP C showed the highest burden (Kruskal–Wallis *H* = 9.526, *P* = 0.009, Dunn’s multiple comparison test, Fig. 1A and G). Notably, TDP A-*C9* cases showed the greatest GCL pathology in the type A subtype despite low neocortical pathology (Fig. 1A and G). GCL pathology did not correlate with frontal or temporal burden (Fig. 1H and I).

Substantial variability in pTDP-43 pathology was evident even within subgroups, with cases of similar disease duration and onset showing variable pathology across cortical regions and hippocampus (Supplementary Table 1).

### HnRNP localization is disrupted and can lead to pathological inclusions in FTLD-TDP

Since TDP-43, an hnRNP family protein, showed variable pathology across FTLD-TDP cases, we examined the expression and localization of additional hnRNPs. We performed semi-quantitative assessments of hnRNP immunostaining in frontal cortex neurons across FTLD subtypes and controls (Supplementary Figs 2–4), with median scores summarized in Supplementary Table 3.

In controls, hnRNP A2/B1, D1/2, H1 and I were predominantly nuclear with minimal cytoplasmic signal (Supplementary Figs 2 and 3). In FTLD-TDP, these showed increased cytoplasmic localization across all subtypes. HnRNP E1/E2 and F were mainly cytoplasmic in controls with hnRNP F showing increased nuclear staining in FTLD. HnRNP M, U and C remained primarily nuclear in both groups, though hnRNP C displayed cytoplasmic staining in controls not observed in disease (Supplementary Figs 2 and 4). HnRNP A1, L, G, R and Q showed both nuclear and cytoplasmic localization without disease-associated shifts. No significant differences were detected between TDP A (sporadic) and TDP A-*C9* cases (Supplementary Figs 2–4).

**Figure 2:**
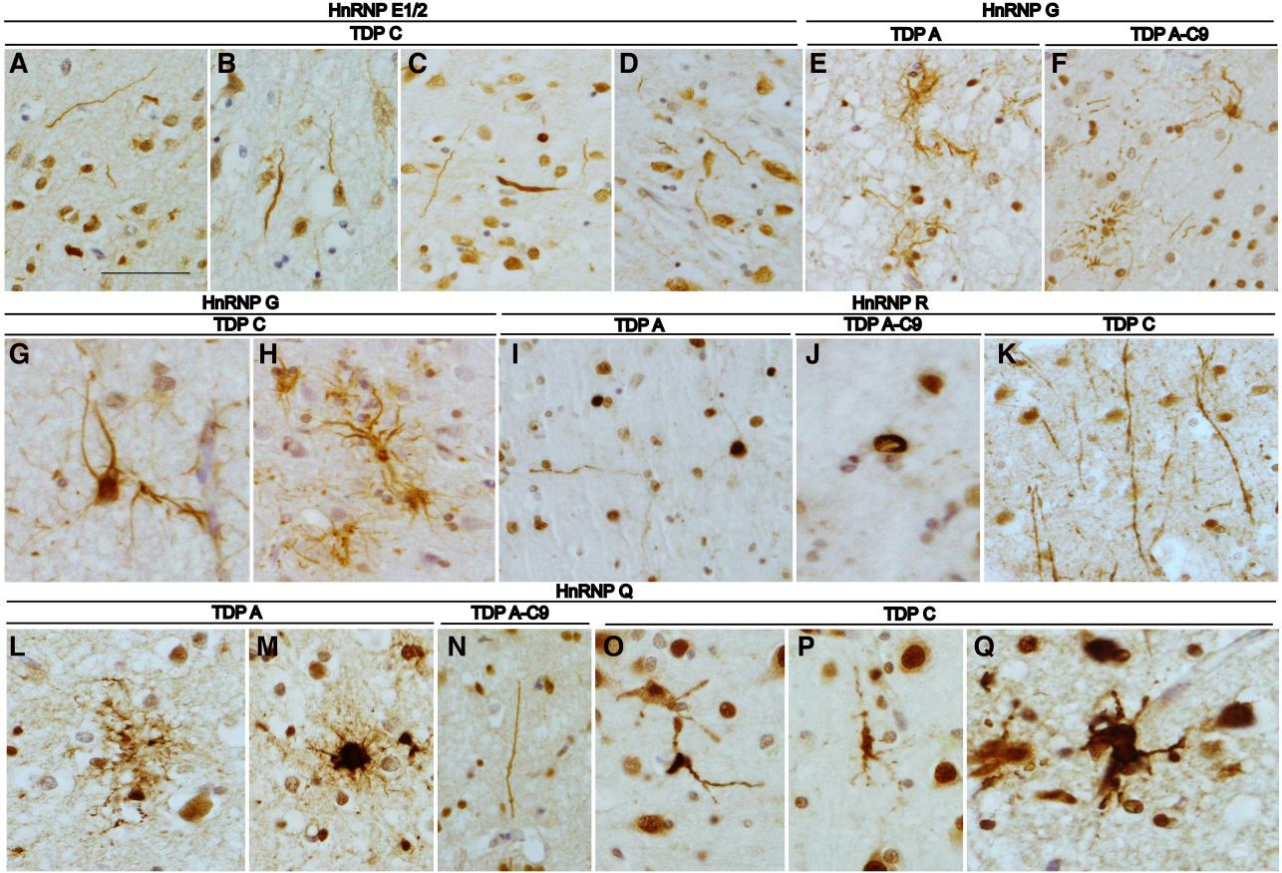
**HnRNP pathologies present in FTLD-TDP subtypes.** HnRNP E is observed in neurites in FTLD-TDP C (**A** and **B**, case 34; **C** and **D**, case 29). HnRNP G accumulation is seen in astrocytes in all subtypes, sporadic TDP A (**E**, case 1), TDP A-*C9* (**F**, case 20) and TDP C (**G**, case 29 and **H**, case 32). HnRNP R accumulation is present in neuronal cytoplasmic inclusions and neuronal processes in sporadic TDP A (**I**, case 5), cytoplasmic inclusions in TDP A-*C9* (**J**, case 26) and in axonal projections in TDP C (**K**, case 34). HnRNP Q accumulation is seen in all FTLD-TDP subtypes although different cellular morphologies are affected. In sporadic TDP A astrocytic profiles show an accumulation of hnRNP Q (**L** and **M**, case 1). In TDP A-*C9*, hnRNP Q is seen in neuritic profiles (**N**, case 19). In TDP C cases, both microglia (**O** and **P**, case 34) and astrocytes (**Q**, case 32) are seen to accumulate hnRNP Q. Scale bar represents 50 µm in **A–F**, **H**, **I**, **K** and 30 µm in **G**, **J**, **L–Q**.

Pathological hnRNP inclusions were detected across FTLD-TDP subtypes with distinct patterns. HnRNP E1/E2 labelled dystrophic neurites in TDP C (Fig. 2A–D). HnRNP G robustly accumulated in astrocytes across subtypes (Fig. 2E–H), with variable staining of cell bodies and processes. HnRNP R accumulated in NCIs and axonal projections in sporadic TDP A (Fig. 2I) and TDP A-*C9* (Fig. 2J) and in axonal projections in TDP C (Fig. 2K), indicating a possible involvement in axonal transport or structural integrity within affected neurons. HnRNP Q showed subtype-dependent patterns: strong astrocytic staining in sporadic TDP A (Fig. 2L and M) and TDP C (Fig. 2Q), neuritic localization in TDP A-*C9* (Fig. 2N) and microglial accumulation in TDP C (Fig. 2O and P), indicating broad glial involvement in this subtype.

### Single-nuclei transcriptomic distribution of FTLD-TDP subtypes compared to control

**Figure 3:**
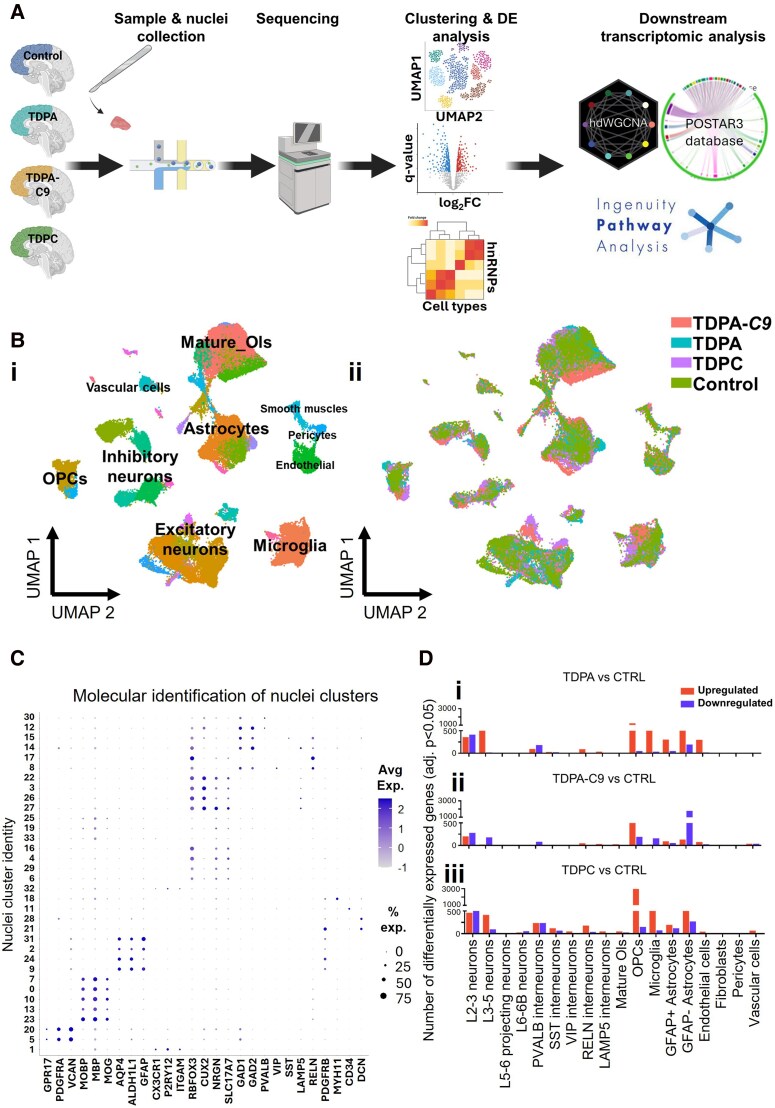
**Workflow and characterization of cell types and molecular profiles in FTLD subtypes:** (**A**) Schematic representation of the experimental workflow. Frontal cortex samples from FTLD-TDP subtypes (TDP A; *n* = 3, TDP A-*C9*; *n* = 3 and TDP C; *n* = 4) and normal controls (NC; *n* = 5) were dissociated into single nuclei, followed by snRNA-seq and downstream computational analysis. The schematic was created in BioRender. Lashley, T. (2026) https://BioRender.com/0r3fle7. (**B**) UMAP plots displaying the clustering of 93 833 single nuclei, (i) annotated into major cell types, including astrocytes, microglia, excitatory neurons, inhibitory neurons, mature oligodendrocytes, OPCs and vascular cells and highlighting nuclei distribution across FTLD subtypes (ii; TDP A, TDP C, TDP A-*C9*) and normal controls. (**C**) Dot plot summarizing the molecular characterization of numbered clusters from 0 to 33. Dot size represents the percentage of cells expressing the marker gene, and colour intensity indicates average expression level. Specific cell cluster assignments are stated in Supplementary Table 4. (**D**) Bar chart indicating total number of significant differentially expressed genes across all neural cell clusters of (i) TDPA, (ii) TDPA-*C9* and (iii) TDPC compared with control. Graphs represent positive and negative log_2_ fold changes as red and blue, respectively, with significance being set at FDR < 0.05 using a Wilcoxon test followed by the Benjamini–Hochberg method. CTRL = healthy controls, Mature Ols = mature oligodendrocytes.

To determine how each cell type in the frontal cortex was affected in FTLD-TDP, we undertook a snRNA-seq study in a subset of sporadic TDP A (*n* = 3), TDP A-*C9* (*n* = 3) cases and TDP C (*n* = 6), along with neurologically normal controls without TDP-43 pathology (*n* = 5; Fig. 3A, Supplementary Table 1). After quality control preprocessing and excluding two samples for not passing checks (two TDP C cases), we recovered a total of 93 833 nuclei across all samples, of which we identified a total of 34 clusters (Fig. 3B (i) and (ii), Supplementary Table 4). Within the 34 clusters sequenced, we identified all the main neural cell types (Fig. 3C). This included seven excitatory neuronal clusters, five distinct inhibitory neuronal clusters (*VIP, SST, PVALB, LAMP5* and *RELN* expressing), as well as glial cells; six oligodendrocyte, two microglial, four astrocytic and two oligodendrocyte precursor cell (OPCs) clusters (Supplementary Table 4). We also collected nuclei from endothelial cells, fibroblasts and pericytes, as well as other cells relating to vasculature (peripheral immune cells, leptomeningeal cells). We began by inferring cortical layer specificity of excitatory neurons through comparative mapping to annotated reference datasets from the CZ CellxGene Discover database and relevant published snRNA-seq datasets.^31,32^ This enabled classification of excitatory neuronal subtypes based on their transcriptomic alignment with known laminar markers. Following this, we identified significant changes in differential gene expression in the L2–3 excitatory neurons, mature oligodendrocytes, OPCs and astrocytes across all FTLD subtypes compared to control, with more significant changes seen in L3–5 in sporadic TDP A and TDP C (Fig. 3D (i) and (iii)).

### Transcriptomic impact on excitatory neurons across FTLD-TDP subtypes

**Figure 4:**
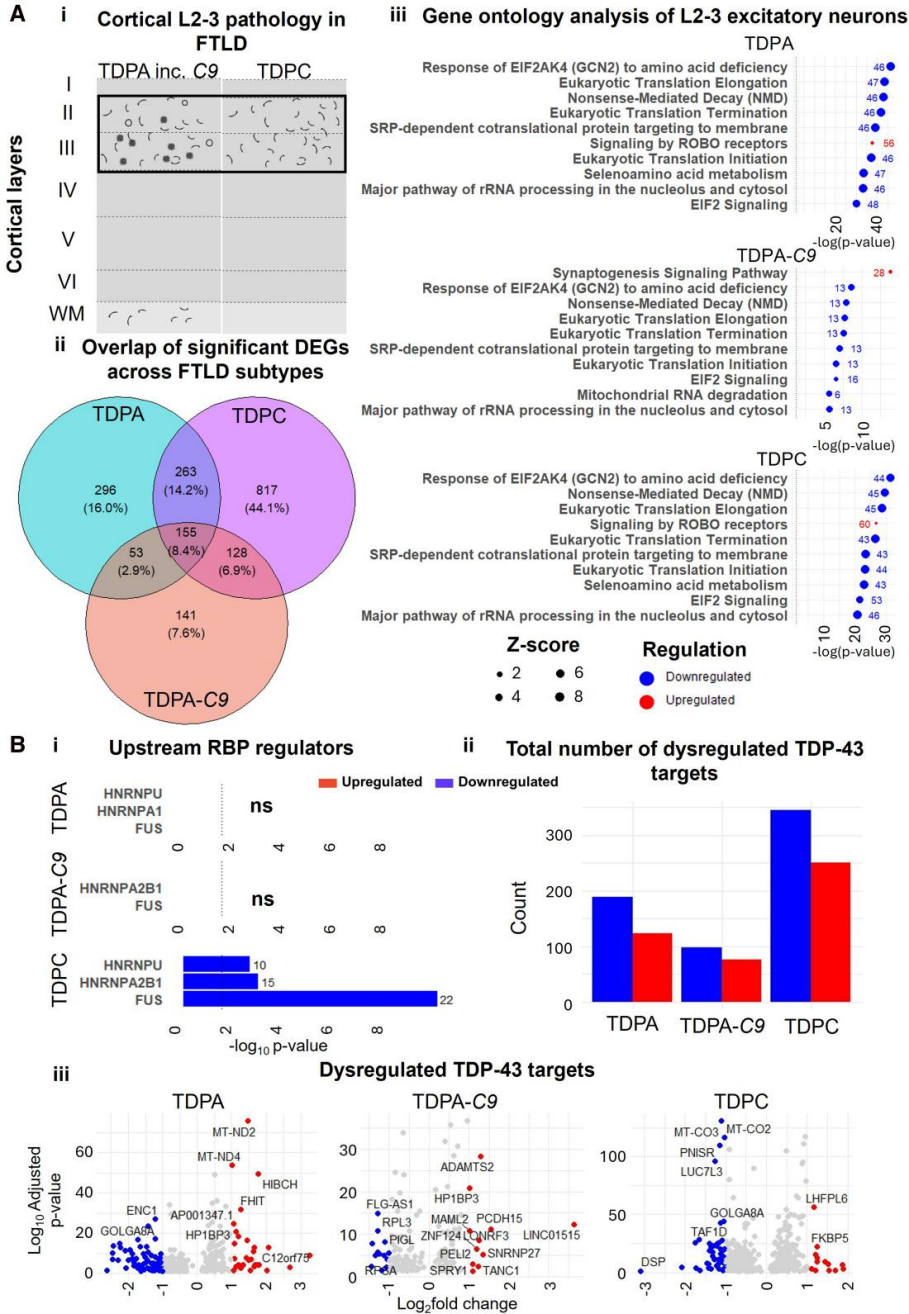
**Characterization of L2–3 excitatory neurons across FTLD-TDP subtypes:** (**A**) (i) Schematic illustration of TDP-43 pathology in the cortex of FTLD-TDP subtype A (TDPA; *n* = 3 and TDPA-*C9*; *n* = 3) and subtype C (TDPC; *n* = 4) cases, with a focus on L2–3 cortical layers. Schematic was created in BioRender. Lashley, T. (2026) https://BioRender.com/0r3fle7. (ii) Venn diagram showing the overlap of significantly DEGs between FTLD-TDP subtypes relative to control in L2–3 excitatory neurons. Total gene counts are indicated, with the percentage of the total DEG pool shown in parentheses. (iii) Dot plot of the top 10 canonical pathways enriched in L2–3 excitatory neurons across FTLD-TDP subtypes compared to control. Pathway significance was determined by right-tailed Fisher’s exact test, with a −log_10_  *P*-value threshold of 1.3 (dotted line). Dot size reflects the predicted activation *z*-score, while colour denotes activation status (red = positive, blue = negative). The number adjacent to each dot indicates the number of DEGs associated with that pathway. (**B**) (i) Upstream regulator analysis focused on hnRNPs across FTLD-TDP subtypes. Regulators are ranked by −log_10_ adjusted *P*-value, with direction of enrichment (up- or down-regulated) highlighted. NS = not significant. (ii) Bar plot showing the total number of significant DEGs overlapping with POSTAR3-derived TDP-43 targets, stratified by expression direction (red = upregulated, blue = downregulated). (iii) Volcano plots of differentially expressed TDP-43 target genes in each FTLD subtype (TDPA, TDPC and TDPA with *C9orf72* mutation) relative to controls. Red and blue dots indicate significantly up- or downregulated genes, respectively, based on −log_10_ adjusted *P*-value and log_2_ fold change. Selected genes of interest are labelled.

L2–3 excitatory neurons were analysed due to their higher vulnerability to TDP-43 pathology in FTLD-TDP (Fig. 4A (i)). All FTLD subtypes shared a core set of DEGs (Fig. 4A (ii); Supplementary Table 4), with greater transcriptional similarity between TDP A and TDP A-*C9*, while TDP C showed a distinct transcriptomic profile and the highest number of unique DEGs (Supplementary Fig. 5).

Canonical pathway analysis revealed subtype-specific alterations in L2–3 neurons (Fig. 4A (iii); Supplementary Table 5). TDP A showed downregulation of translation-related pathways, including EIF2AK4 response, translation initiation/elongation/termination and nonsense-mediated decay, alongside reduced SRP-dependent targeting, selenocysteine metabolism and rRNA processing. ROBO signalling was upregulated. TDP A-*C9* shared downregulation of EIF2AK4 and translation pathways but uniquely showed upregulation of synaptogenesis signalling, with concurrent downregulation of mitochondrial RNA degradation and rRNA processing. TDP C displayed a similar profile to TDP A, with downregulation of translation-associated pathways and upregulation of ROBO signalling, alongside marked suppression of selenocysteine metabolism.

*TARDBP* was not differentially expressed in L2–3 neurons. Upstream regulator analysis identified predicted disruption of RBPs including *HNRNPA2B1*, *HNRNPU* and *FUS*, reaching significance only in TDP C (Fig. 4B (i)). Analysis of POSTAR3 TDP-43 targets showed widespread dysregulation, most pronounced in TDP C and moderate in TDP A, with fewer dysregulated targets in TDP A-*C9* (Fig. 4B (ii)). Gene-level changes included upregulation of *MT-ND2* and *MT-ND4* in TDP A, and downregulation of *MT-CO2* and *PNISR* in TDP C (Fig. 4B (iii).

**Figure 5:**
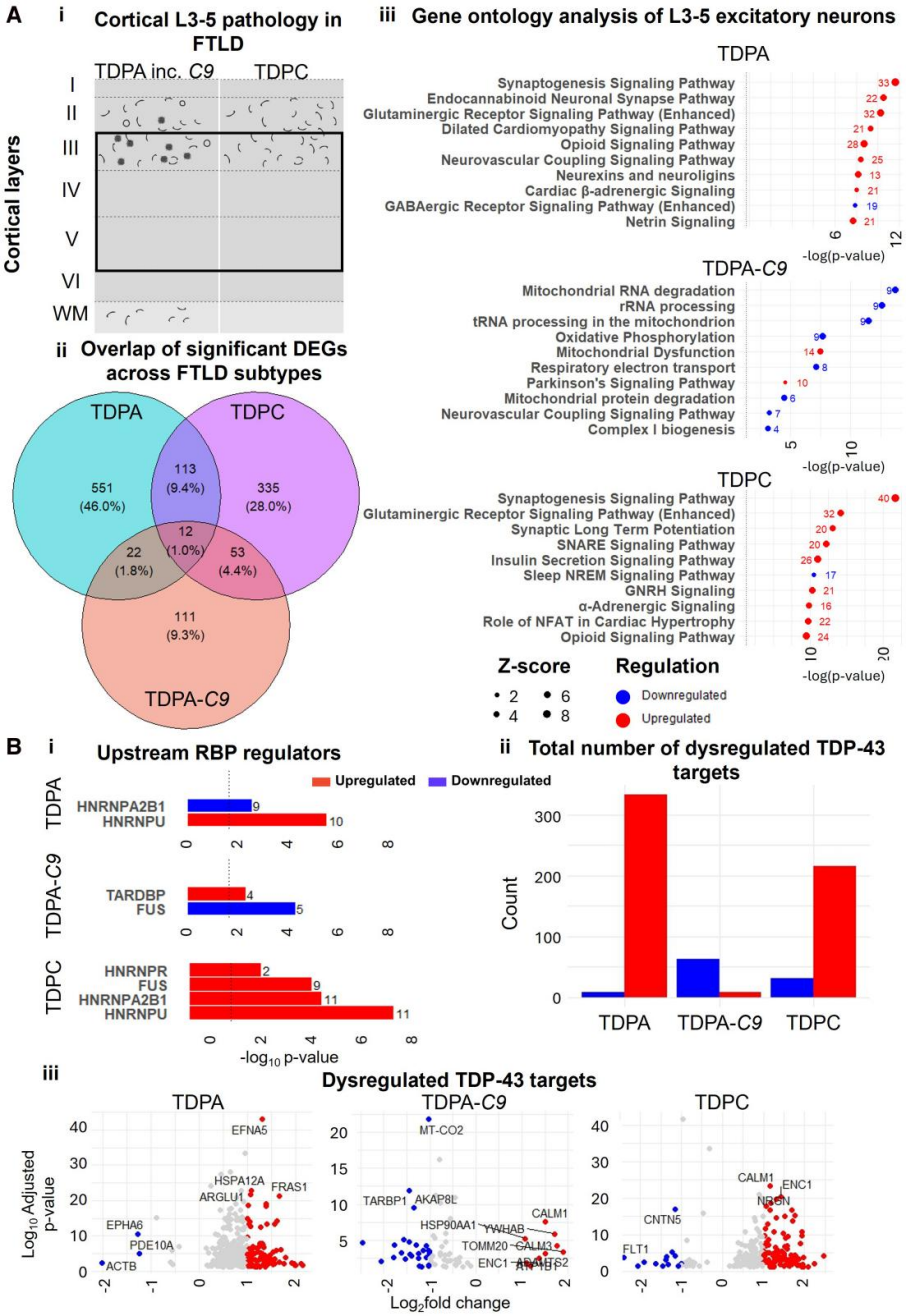
**Characterization of L3–5 excitatory neurons across FTLD-TDP subtypes:** (**A**) (i) Schematic illustration of TDP-43 pathology in the cortex of FTLD-TDP subtype A (TDPA; *n* = 3 and TDPA-*C9*; *n* = 3) and subtype C (TDPC; *n* = 4) cases, with a focus on L3–5 cortical layers.^36^ Schematic was created in BioRender. Lashley, T. (2026) https://BioRender.com/0r3fle7. (ii) Venn diagram showing the overlap of significantly DEGs between FTLD-TDP subtypes relative to control in L3–5 excitatory neurons. Total gene counts are indicated, with the percentage of the total DEG pool shown in parentheses. (iii) Dot plot of the top 10 canonical pathways enriched in L3–5 excitatory neurons across FTLD-TDP subtypes compared to control. Pathway significance was determined by right-tailed Fisher’s exact test, with a −log_10_  *P*-value threshold of 1.3 (dotted line). Dot size reflects the predicted activation z-score, while colour denotes activation status (red = positive, blue = negative). The number adjacent to each dot indicates the number of DEGs associated with that pathway. (**B**) (i) Upstream regulator analysis focused on hnRNPs across FTLD-TDP subtypes. Regulators are ranked by −log_10_ adjusted *P*-value derived from right-tailed Fisher’s exact test, with direction of enrichment (up- or down-regulated) highlighted. NS = not significant. (ii) Bar plot showing the total number of significant DEGs overlapping with POSTAR3-derived TDP-43 targets, stratified by expression direction (red = upregulated, blue = downregulated). (iii) Volcano plots of differentially expressed TDP-43 target genes in each FTLD subtype (TDPA, TDPC and TDPA with *C9orf72* mutation) relative to controls. Red and blue dots indicate significantly up- or downregulated genes, respectively, based on −log_10_ adjusted *P*-value and log_2_ fold change using the Wilcoxon Rank-Sum test. Selected genes of interest are labelled..

In L3–5 excitatory neurons, where TDP-43 pathology was less abundant (Fig. 5A (i)), fewer shared DEGs were observed across subtypes (Fig. 5A (ii)). TDP A was transcriptionally distinct, while TDP A-*C9* and TDP C showed greater overlap (Supplementary Fig. 5). Pathway analysis demonstrated subtype-specific signalling changes (Fig. 5A (iii); Supplementary Table 5). TDP A showed upregulation of synaptogenesis, endocannabinoid, glutamatergic, GABAergic, Neurexin/Neuroligin, Netrin and neurovascular coupling pathways. TDP A-*C9* showed downregulation of mitochondrial and bioenergetic pathways, including oxidative phosphorylation, respiratory electron transport, complex I biogenesis, tRNA processing and mitochondrial protein degradation. TDP C exhibited upregulation of synaptic long-term potentiation, SNARE signalling, opioid and glutamatergic receptor pathways.

Upstream regulator analysis in L3–5 neurons showed stronger enrichment of RBPs than in L2–3 (Fig. 5B (i)), including *HNRNPA2B1*, *HNRNPU*, *TARDBP*, *HNRNPR* and *FUS*, with subtype-specific patterns. Dysregulation of TDP-43 targets was greatest in TDP A, reduced in TDP A-*C9* and moderately increased in TDP C (Fig. 5B (ii)). Gene-level changes included upregulation of *EFNA5* and *FRAS1* in TDP A, downregulation of *MT-CO2* and *TARBP1* in TDP A-*C9*, and upregulation of *CALM1, NRGN* and *ENC1* in TDP C (Fig. 5B (iii)).

### Differential gene expression analysis reveals a predominant alteration of multiple hnRNPs in glial nuclei across FTLD subtypes

**Figure 6:**
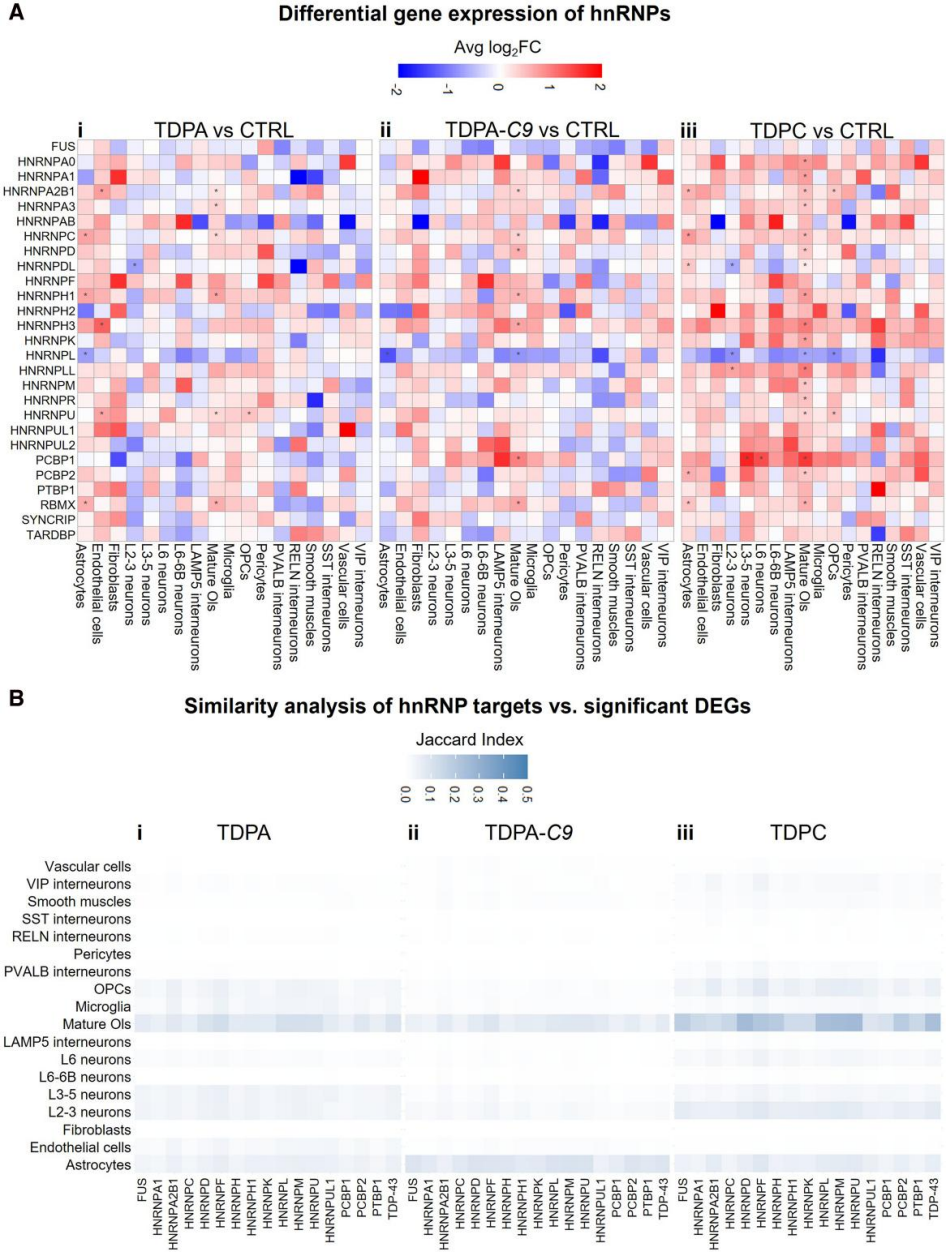
**Transcriptional dysregulation of hnRNPs in glial clusters in FTLD subtypes:** (**A**) Heatmaps showing the differential gene expression of FTLD (i, TDP A; *n* = 3, ii, TDP A-*C9*; *n* = 3 and iii, TDP C; *n* = 4) cases against controls. Red- and blue-coloured boxes indicate a positive and negative log_2_ fold change, respectively, with an asterisk indicating an FDR of <0.05. FDR was derived using Wilcoxon test followed by the Benjamini–Hochberg method (**B**) Similarity analysis heatmap of hnRNP targets with significant differential gene expression profile across all neural nuclei clusters of (i) TDP A, (ii) TDP A-*C9* and (iii) TDP C. Higher Jaccard indices are indicated by more solid-coloured boxes.

Expression of hnRNP genes across cell types was analysed to assess hnRNP network alterations across FTLD subtypes (Fig. 6A). *TARDBP* expression was not significantly altered in any subtype or cluster. In TDP A versus controls, increased expression of multiple *HNRNP* genes was observed primarily in mature oligodendrocytes and astrocytes, with smaller changes in endothelial cells and reduced *HNRNPL* in astrocytes (Fig. 6A (i)). Neuronal changes were limited to reduced *HNRNPDL* in L2–3 excitatory neurons. In TDP A-*C9*, hnRNP changes were again concentrated in oligodendrocytes and astrocytes, with marked downregulation of *HNRNPL* (Fig. 6A (ii)). Consistent patterns were observed across datasets, with oligodendrocytes showing widespread upregulation of HNRNP genes except *HNRNPL* and *HNRNPDL*, which were downregulated across astrocytes, OPCs, SST interneurons and excitatory neurons (L2–3, L3–5 and L6; Supplementary Fig. 6). In TDP C, most *HNRNP* genes were upregulated in mature oligodendrocytes, with additional upregulation of *HNRNPA2B1, HNRNPC, HNRNPDL, PCBP2* and *RBMX* in astrocytes and *HNRNPA2B1* and *HNRNPU* in OPCs (Fig. 6A (iii)). *HNRNPL* was downregulated in L2–3 neurons, mature oligodendrocytes and OPCs.

Overlap between hnRNP targets across clusters was assessed using Jaccard similarity analysis (Fig. 6B). Increased overlap was observed between oligodendrocytes, astrocytes and L2–3 excitatory neurons across hnRNP targets (Fig. 6B (i) and (ii)), with the strongest overlap in TDP C, particularly involving astrocytes and OPCs (Fig. 6B (iii)). Oligodendrocytes in TDP C also showed higher Jaccard indices with hnRNPs D, L, M and U, and TDP-43 targets.

**Figure 7:**
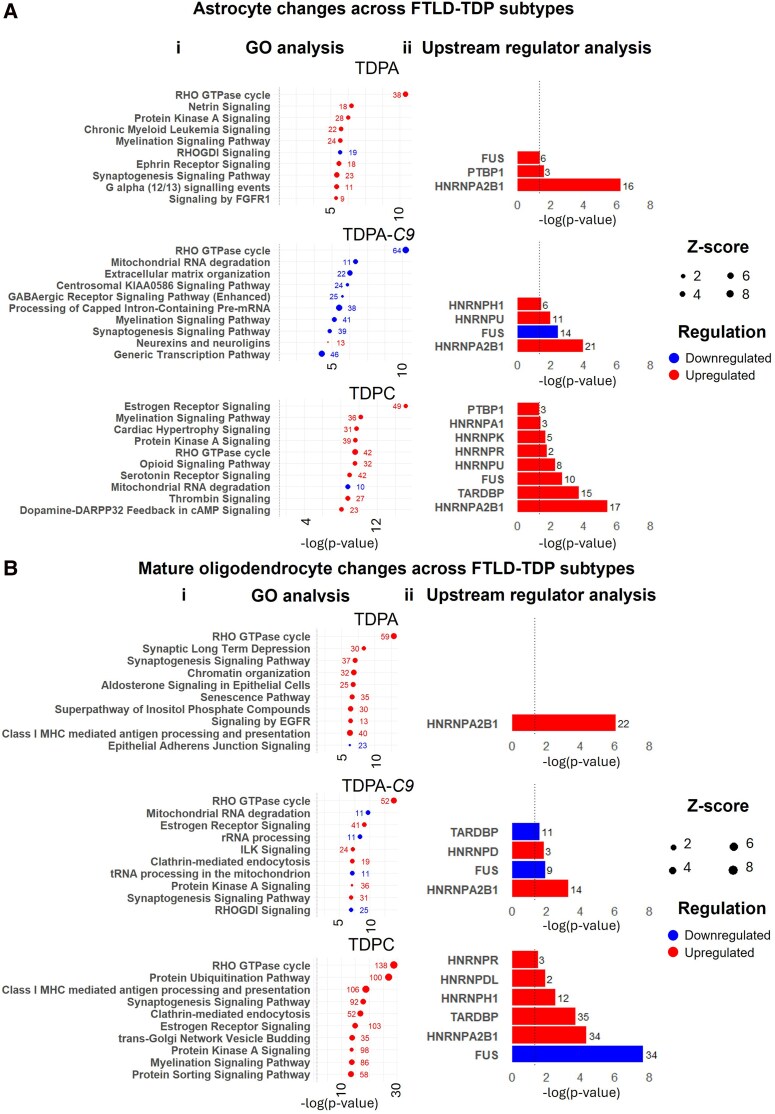
**Characterization of astrocytes and oligodendrocytes across FTLD-TDP subtypes:** (**A**) (i) Dot plots illustrating the top 10 canonical pathways significantly enriched in astrocytes from FTLD-TDP cases relative to control. Enrichment was assessed using a right-tailed Fisher’s exact test, with a −log_10_  *P*-value significance threshold of 1.3 (dotted line). Dot size corresponds to the activation *z*-score, and colour indicates activation state (red = activated; blue = inhibited). Numbers next to each dot represent the count of DEGs mapping to each pathway. (ii) Upstream regulator analysis constrained to hnRNP family members. Regulators are ranked by statistical significance (−log_10_ adjusted *P*-value), and direction of enrichment is indicated using a right-tailed Fisher’s exact test. (**B**) (i) Dot plots illustrating the top 10 canonical pathways significantly enriched in mature oligodendrocytes from FTLD-TDP cases relative to control. Enrichment was assessed using a right-tailed Fisher’s exact test, with a −log_10_  *P*-value significance threshold of 1.3 (dotted line using a right-tailed Fisher’s exact test). Dot size corresponds to the activation *z*-score, and colour indicates activation state (red = activated; blue = inhibited). Numbers next to each dot represent the count of DEGs mapping to each pathway. (ii) Upstream regulator analysis constrained to hnRNP family members. Regulators are ranked by statistical significance (−log_10_ adjusted *P*-value), and direction of enrichment is indicated. CTRL = healthy controls..

In astrocytes, pathway analysis revealed subtype-specific enrichment profiles (Fig. 7A (i); Supplementary Table 5). TDP A was enriched for synaptic and axon guidance pathways, TDP A-*C9* for mitochondrial and transcriptional processes, and TDP C for neuromodulatory signalling. Upstream regulator analysis identified distinct RBP enrichments across subtypes, with *HNRNPA2B1* significantly increased in all groups and the broadest RBP enrichment in TDP C, including *TARDBP, FUS, HNRNPU, HNRNPR, HNRNPK, HNRNPA1* and *PTBP1* (Fig. 7A (ii)).

In mature oligodendrocytes, pathway analysis showed enrichment of cytoskeletal, chromatin and immune-related pathways in TDP A, RNA processing and mitochondrial dysfunction in TDP A-*C9*, and protein degradation, sorting and antigen presentation in TDP C (Fig. 7B (i)). Upstream regulator analysis again showed the broadest RBP involvement in TDP C, including *TARDBP, FUS* and multiple *HNRNPs*, while both TDP A groups showed a more restricted profile dominated by *HNRNPA2B1* (Fig. 7B (ii)).

### Astrocytes and oligodendrocytes transcriptionally drive splice alterations in FTLD-TDP

**Figure 8:**
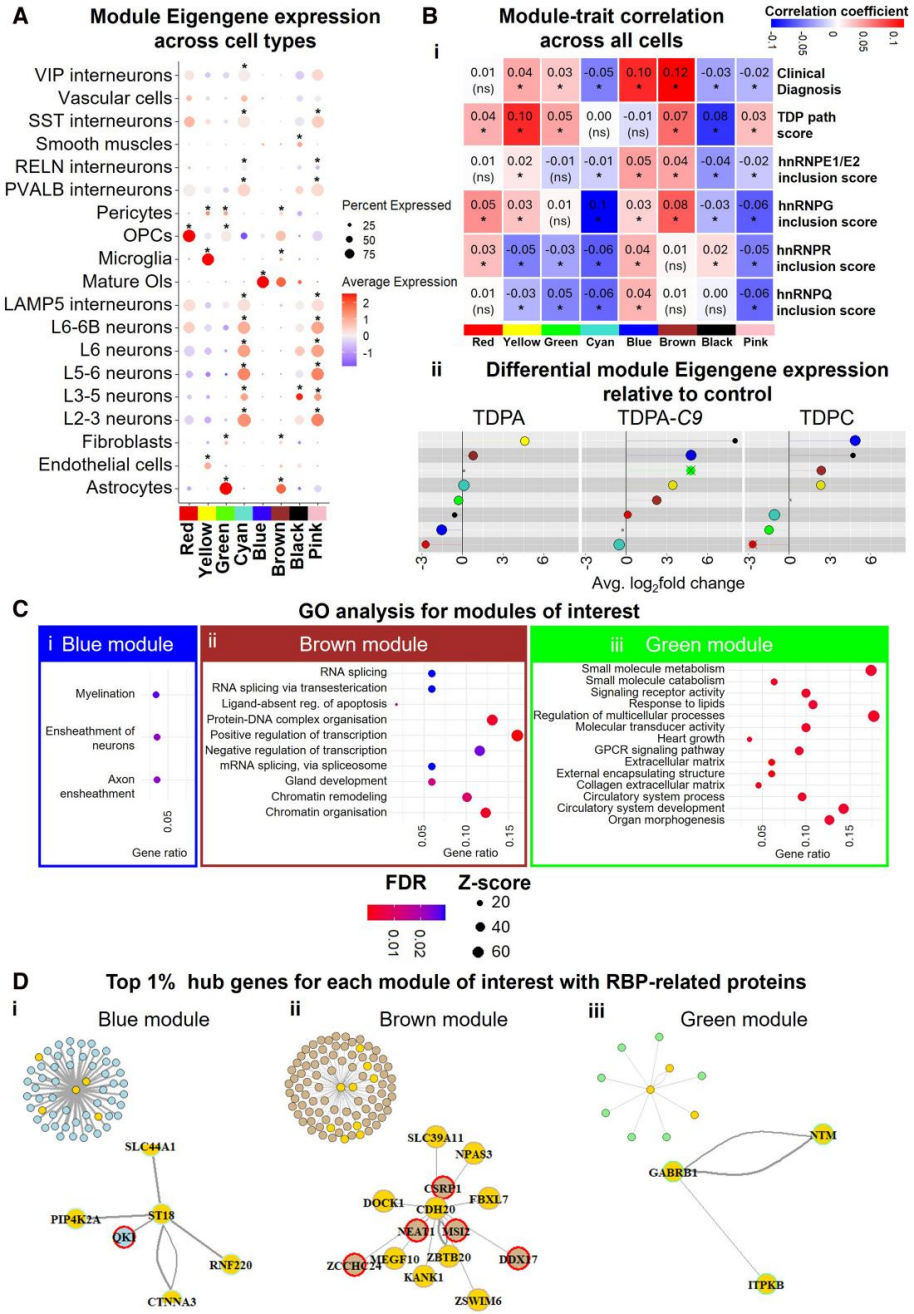
**High-dimensional weighted gene co-expression network analysis of FTLD-TDP subtypes:** (**A**) Dot plot summarizing MEs identified through hdWGCNA, annotated by their enrichment across distinct cell types from the snRNA-seq dataset of FTLD-TDP subtype A (TDPA; *n* = 3 and TDPA-*C9*; *n* = 3) and subtype C (TDPC; *n* = 4) cases. Cell-type enrichment was assessed by testing over-representation of each module’s gene set among cell-type marker genes using a hypergeometric test, with the WGCNA gene set used as the background universe; *P*-values were Benjamini–Hochberg corrected, and significant enrichments (FDR < 0.05) are indicated on the plot. (**B**) (i) Module-trait correlation matrix showing the relationship between module eigengene expression and clinical/pathological variables, including disease classification, TDP-43 pathology score and hnRNP (E1/E2, G, R, Q) inclusion scores. Pearson’s correlation coefficients are visualized by colour intensity (red = positive, blue = negative); significance assessed using two-tailed *t*-tests with Bonferroni correction for multiple testing (FDR is shown in parentheses with values <0.05 indicated with an asterisk). (ii) Lollipop plots depicting changes in module eigengene expression for each FTLD-TDP subtype relative to control. Modules are plotted by average log_2_ fold change; non-significant differences (FDR ≥ 0.05, Wilcoxon test with Benjamini–Hochberg correction) are marked with a cross. (**C**) GO enrichment analysis of selected modules: (i) blue, (ii) brown and (iii) green. Dot plots display the top-ranked pathways by enrichment significance, with dot size indicating the number of genes contributing to each pathway. (**D**) Hub gene analysis for the same modules: (i) blue, (ii) brown and (iii) green. The top 1% most interconnected genes within each module are shown, with the top 10% hub network plots in smaller inserts for each module. Genes associated with RNA-binding, splicing or transport functions are outlined in red. Mature Ols = mature oligodendrocytes.

To explore disease-associated transcriptomic changes in the astrocytes and oligodendrocytes in more detail, and to explore the transcriptional heterogeneity underlying FTLD-TDP, we applied hdWGCNA. The analysis identified eight distinct eigengene co-expression modules, each characterized by unique transcriptional signatures within our dataset (Fig. 8A). The green and blue modules were significantly expressed in astrocytes and mature oligodendrocytes, respectively, while the brown module was expressed in both but reached significance only in astrocytes.

Module-trait correlation analysis showed that the green, brown and blue modules were significantly associated with disease status (control versus FTLD; Fig. 8B (i)). These modules also correlated with inclusion burden for TDP-43 and multiple hnRNP proteins. The brown and green modules showed positive correlations with both TDP-43 pathology scores, while the brown and blue modules correlated with hnRNP E1/E2 and hnRNP G inclusion scores. The blue module additionally correlated with hnRNP R and hnRNP Q inclusion scores (Fig. 8B (i)).

Differential module eigengene analysis revealed consistent upregulation of the brown module across all FTLD subtypes (Fig. 8B (ii)). The blue module was upregulated in TDP A-*C9* and TDP C but downregulated in TDP A, while the green module was slightly downregulated in TDP A and TDP C and upregulated in TDP A-*C9* (Fig. 8B (ii)).

Gene ontology enrichment analysis showed subtype-relevant functional signatures for cell type-enriched modules (Fig. 8C). The oligodendrocyte-enriched blue module was associated with myelination pathways (Fig. 8C (i)). The astrocyte-enriched brown module was associated with RNA splicing, transcriptional regulation and chromatin organization (Fig. 8C (ii)). The astrocyte-enriched green module was associated with small-molecule processing, receptor activity and lipid responses (Fig. 8C (iii)).

Hub gene analysis identified key drivers of module expression (Fig. 8D; Supplementary Table 6). In the blue module, top hub genes included *ST18, CTNNA3, PIP4K2, SLC44A1* and *RNF220*, with *QKI* present among the top 10% hubs (Fig. 8D (i)). The brown module showed enrichment of hub genes including *CDH20, ZBTB20, MEGF10, ZSWIM6, NPAS3, KANK1, SLC39A1, DOCK1 a*nd *FBXL7*, alongside RNA-related genes *ZCCHC2, DDX17, CSRP1, MSI2* and *NEAT1* (Fig. 8D (ii)). In the green module, *GABRB1, NTM* and *ITPKB* were identified as top hub genes (Fig. 8D (iii)).

## Discussion

Our findings demonstrate that multiple hnRNP family members contribute to FTLD and highlight the importance of examining non-neuronal cell types. While TDP-43 remains the most studied hnRNP, other hnRNPs show subtype- and region-specific pathology and transcriptomic changes. In the frontal cortex, several hnRNPs displayed altered localization, including cytoplasmic enrichment without clear inclusion formation, supporting the idea that hnRNPs act as a coordinated network in which dysfunction of one member can affect others.^25^

In the hippocampal DG, a region frequently affected in FTLD, we confirm that pTDP-43 pathology is restricted to NCIs. Type C cases, clinically linked to semantic dementia,^5^ show the highest NCI burden in granule cells, whereas sporadic type A, typically associated with bvFTD,^5^ shows the lowest. Importantly, DG pathology does not correlate with frontal or temporal cortical inclusion burden. It is not clear whether hippocampal pathology burden contributes to disease progression synergistically with what is occurring in the frontal/temporal cortical regions, or whether it reflects a parallel, region-specific vulnerability. Future studies correlating DG pathology with longitudinal clinical outcomes could clarify its functional significance in FTLD.

Investigation of hnRNP immunohistochemistry across sporadic (TDP A, TDP C) and genetic (TDP A-*C9*) subtypes revealed mislocalization of hnRNP R and Q, previously seen in FTLD-FUS,^44,45^ and their accumulation in axonal/neuritic projections in FTLD-TDP. We also confirm hnRNP E1/2 in dystrophic neurites typical of TDP C.^21,22^ Although many hnRNPs did not form classic inclusions, several showed increased cytoplasmic localization, which may impair their function.

We observed increased hnRNP immunoreactivity in non-neuronal cells. Astrocytic hnRNP G was elevated across all FTLD subtypes; hnRNP G is involved in DNA damage responses and is upregulated in astrocytes after injury.^46-48^ However, interpretation of transcriptional changes in *RBMX*, the X-linked gene encoding hnRNP G, should be approached with caution, as the cohort size in this study was insufficient to allow sex-stratified analyses. HnRNP R and Q showed subtype-specific aggregation; hnRNP Q also accumulated in astrocytes (TDP A, C) and microglia (TDP C). Both proteins modulate TDP-43 toxicity, with *SYNCRIP* knockdown rescuing TDP-43-induced toxicity.^49^ HnRNP Q is also dysregulated in ALS, showing altered localization in sporadic versus *C9orf72* cases.^50^ Both hnRNP Q and R appear in FTLD-FUS inclusions,^44^ though their functional relationship remains unclear. Their redistribution in glia may represent broader molecular disturbances, consistent with predicted dysregulated FUS activity in glial populations.^51^

SnRNA-seq revealed that hnRNP network changes at the protein level do not always mirror transcriptomic alterations. *HNRNPL* was the only hnRNP significantly altered across at least one cellular cluster in all subtypes, validated in the Gittings dataset.^31^ HnRNP L stabilizes transcripts prone to NMD,^52,53^ regulates *UNC13A* splicing^54^ and increases in response to TDP-43 aggregation.^55^ Despite transcriptomic changes, no abnormal hnRNP L localization was observed at the protein level. Similarly, *TARDBP* expression was unchanged despite clear pathological aggregation. These discrepancies emphasize that disease-associated transcriptional changes can be subtle, cell-type-specific and easily obscured in bulk tissue, underscoring the value of single-cell approaches for resolving hnRNP network perturbations.

Upstream analysis predicted reduced FUS activity in L2–3 neurons across FTLD-TDP, without evidence of FUS mislocalization, potentially reflecting secondary effects of TDP-43 pathology. Structural differences between TDP-43 inclusion types may contribute: type A inclusions are more insoluble, sequester more RBPs, and have stronger biochemical effects than type C neurites.^56-59^ Cryo-EM studies show type A fibrils disrupt RNA-protein interactions and nucleocytoplasmic transport via TNPO1.^60^ HnRNP target analysis showed broader but milder RNA network disruption in TDP C, whereas TDP A inclusions appear to drive more localized dysfunction via RBP sequestration.^56-59^

L2–3 excitatory neurons showed prominent translational and stress-response deficits, including reduced EIF2AK4 (GCN2) activity and suppression of core protein synthesis pathways. These features were most evident in sporadic TDP A and C cases, consistent with the heightened vulnerability of superficial cortical layers. Given EIF2AK4’s role in translation under stress,^61^ these results suggest impaired proteostasis. Although EIF2AK4 dysregulation has been described in *C9orf72* models,^62^ our findings expand its relevance to sporadic FTLD. In contrast, L3–5 neurons showed more variable subtype-specific alterations. In TDP A, these neurons showed upregulated synaptic and neurovascular signalling, including glutamatergic, GABAergic, endocannabinoid and neurexin/neuroligin pathways, suggesting synaptic remodelling and altered excitation-inhibition balance.^63^ Increased ROBO signalling in TDP A and C may reflect responses to axonal injury, consistent with ALS studies.^64-66^

Together, these findings suggest layer-specific TDP-43-associated dysfunction: L2–3 neurons show weaker enrichment of other RBPs and greater disruption of TDP-43 target transcripts in TDP C, while L3–5 neurons show stronger RNA dysregulation and subtype-specific RBP involvement.

Glial analysis further highlighted hnRNP network disruptions. TDP C cases showed significant oligodendrocyte transcriptional changes, consistent with independent datasets.^31,67^ Previous FTLD-*GRN* snRNA-seq studies also report major astrocytic changes.^68^ These findings indicate substantial glial contributions to FTLD-TDP and suggest that hnRNP disruptions converge with broader astrocytic dysfunction. Upstream analysis identified hnRNP A2B1 dysregulation in astrocytes and oligodendrocytes; hnRNP A2B1 regulates RNA splicing and transport essential for glial function and may respond to neuronal TDP-43 pathology.^19,69^ Co-expression network analysis revealed glial-enriched modules strongly associated with diagnosis, consistent with recent work.^70^ Key regulators included *NEAT1, MSI2, DDX17* and *QKI*, with *QKI* serving as an oligodendrocyte hub linking RNA processing deficits to myelination abnormalities.^71-74^ Astrocytic modules lacked classical RNA-processing hubs but showed disrupted small-molecule and lipid metabolism. Age also influences hnRNP expression in a cell-specific manner, contributing to mis-splicing and RBP mislocalization.^75,76^ Comparing age-matched controls and FTLD cases revealed glial-specific transcriptomic changes that may be disease-relevant even without corresponding protein alterations. This aligns with recent work showing that cryptic exon expression correlates more strongly with itself than with visible TDP-43 pathology.^77^ TDP-43-positive oligodendrocyte inclusions are well established,^78^ with newer evidence highlighting TDP-43’s role in oligodendrocyte survival and myelination.^79,80^ Glial dysfunction may therefore precede or exacerbate neuronal pathology, though this cannot be confirmed in *post-mortem* tissue. Larger cohorts will be required to link glial transcriptomic signatures with white-matter TDP-43 burden in a subtype-specific manner.

While some transcriptomic changes likely reflect downstream effects (e.g. mitochondrial stress),^31,81^ others, particularly those involving RNA or synaptic signalling pathways, may result directly from TDP-43 mislocalization, especially in L2–3 neurons. TDP A-*C9* cases in this cohort had a lower mean age at death than the other subtypes. Consequently, some of the differences observed in this subtype may partly reflect age-related molecular changes, which are intrinsically linked to disease mechanisms in *C9orf72*-associated FTLD. Indeed, *C9* carriers typically show a significantly shorter disease duration than sporadic or other genetic forms of FTLD,^82^ and this earlier clinical trajectory may amplify age-sensitive alterations in hnRNP expression and downstream RNA processing. Cohort size also limits subtype-specific or sex-stratified conclusions, but concordance across independent datasets supports the robustness of our findings.

In conclusion, our study characterizes FTLD subtype-specific transcriptomic alterations using snRNA-seq and demonstrates pathological changes across the hnRNP network via histology. We highlight changes in hnRNP expression at both transcript and protein levels, particularly within oligodendrocyte lineages, and underscore the importance of including non-neuronal cell types and diverse hnRNPs in investigations of FTLD pathogenesis.

## Supplementary Material

fcag197_Supplementary_Data

## Data Availability

The raw and pre-processed snRNA-seq data in this publication have been deposited in NCBI’s Gene Expression Omnibus and are accessible through GEO series accession number GSE288106. Other snRNA-seq datasets used for confirmatory analysis were obtained from GEO (GSE219281) and Synapse (syn45351388).

## References

[1] Neary D, Snowden JS, Gustafson L, et al Frontotemporal lobar degeneration: A consensus on clinical diagnostic criteria. Neurology. 1998;51(6):1546–1554. 9855500 10.1212/wnl.51.6.1546

[2] Freischmidt A, Müller K, Ludolph AC, Weishaupt JH, Andersen PM. Association of mutations in TBK1 with sporadic and familial amyotrophic lateral sclerosis and frontotemporal dementia. JAMA Neurol. 2017;74(1):110–113. 27892983 10.1001/jamaneurol.2016.3712

[3] Rohrer JD, Guerreiro R, Vandrovcova J, et al The heritability and genetics of frontotemporal lobar degeneration. Neurology. 2009;73(18):1451–1456. 19884572 10.1212/WNL.0b013e3181bf997a PMC2779007

[4] Rohrer JD, Isaacs AM, Mizielinska S, et al C9orf72 expansions in frontotemporal dementia and amyotrophic lateral sclerosis. Lancet Neurol. 2015;14(3):291–301. 25638642 10.1016/S1474-4422(14)70233-9

[5] Lashley T, Rohrer JD, Mead S, Revesz T. Review: An update on clinical, genetic and pathological aspects of frontotemporal lobar degenerations. Neuropathol Appl Neurobiol. 2015;41(7):858–881. 26041104 10.1111/nan.12250

[6] Neumann M, Sampathu DM, Kwong LK, et al Ubiquitinated TDP-43 in frontotemporal lobar degeneration and amyotrophic lateral sclerosis. Science. 2006;314(5796):130–133. 17023659 10.1126/science.1134108

[7] Mackenzie IRA, Neumann M, Baborie A, et al A harmonized classification system for FTLD-TDP pathology. Acta Neuropathol. 2011;122(1):111–113. 21644037 10.1007/s00401-011-0845-8 PMC3285143

[8] Lee EB, Porta S, Michael Baer G, et al Expansion of the classification of FTLD-TDP: Distinct pathology associated with rapidly progressive frontotemporal degeneration. Acta Neuropathol. 2017;134(1):65–78. 28130640 10.1007/s00401-017-1679-9 PMC5521959

[9] Romano M, Buratti E, Romano G, et al Evolutionarily conserved heterogeneous nuclear ribonucleoprotein (hnRNP) A/B proteins functionally interact with human and Drosophila TAR DNA-binding protein 43 (TDP-43). J Biol Chem. 2014;289(10):7121–7130. 24492607 10.1074/jbc.M114.548859 PMC3945372

[10] Polymenidou M, Lagier-Tourenne C, Hutt KR, et al Long pre-mRNA depletion and RNA missplicing contribute to neuronal vulnerability from loss of TDP-43. Nat Neurosci. 2011;14(4):459–468. 21358643 10.1038/nn.2779 PMC3094729

[11] Tollervey JR, Curk T, Rogelj B, et al Characterizing the RNA targets and position-dependent splicing regulation by TDP-43. Nat Neurosci. 2011;14(4):452–458. 21358640 10.1038/nn.2778 PMC3108889

[12] Piñol-Roma S, Choi YD, Matunis MJ, Dreyfuss G. Immunopurification of heterogeneous nuclear ribonucleoprotein particles reveals an assortment of RNA-binding proteins. Genes Dev. 1988;2(2):215–227. 3129338 10.1101/gad.2.2.215

[13] Geuens T, Bouhy D, Timmerman V. The hnRNP family: Insights into their role in health and disease. Hum Genet. 2016;135(8):851–867. 27215579 10.1007/s00439-016-1683-5 PMC4947485

[14] Dreyfuss G, Matunis MJ, Piñol-Roma S, Burd CG. hnRNP proteins and the biogenesis of mRNA. Annu Rev Biochem. 1993;62:289–321. 8352591 10.1146/annurev.bi.62.070193.001445

[15] Roy R, Durie D, Li H, et al hnRNPA1 couples nuclear export and translation of specific mRNAs downstream of FGF-2/S6K2 signalling. Nucleic Acids Res. 2014;42(20):12483–12497. 25324306 10.1093/nar/gku953 PMC4227786

[16] Busch A, Hertel KJ. Evolution of SR protein and hnRNP splicing regulatory factors. Wiley Interdiscip Rev RNA. 2012;3(1):1–12. 21898828 10.1002/wrna.100 PMC3235224

[17] Bekenstein U, Soreq H. Heterogeneous nuclear ribonucleoprotein A1 in health and neurodegenerative disease: From structural insights to post-transcriptional regulatory roles. Mol Cell Neurosci. 2013;56:436–446. 23247072 10.1016/j.mcn.2012.12.002

[18] Honda H, Hamasaki H, Wakamiya T, et al Loss of hnRNPA1 in ALS spinal cord motor neurons with TDP-43-positive inclusions. Neuropathology. 2015;35(1):37–43. 25338872 10.1111/neup.12153

[19] Martinez FJ, Pratt GA, Van Nostrand EL, et al Protein-RNA networks regulated by normal and ALS-associated mutant HNRNPA2B1 in the nervous system. Neuron. 2016;92(4):780–795. 27773581 10.1016/j.neuron.2016.09.050 PMC5123850

[20] Jovičić A, Paul JW, Gitler AD. Nuclear transport dysfunction: A common theme in amyotrophic lateral sclerosis and frontotemporal dementia. J Neurochem. 2016;138(Suppl 1):134–144. 27087014 10.1111/jnc.13642

[21] Kattuah W, Rogelj B, King A, Shaw CE, Hortobágyi T, Troakes C. Heterogeneous nuclear ribonucleoprotein E2 (hnRNP E2) is a component of TDP-43 aggregates specifically in the A and C pathological subtypes of frontotemporal lobar degeneration. Front Neurosci. 2019;13:551. 31213972 10.3389/fnins.2019.00551 PMC6558155

[22] Davidson YS, Robinson AC, Flood L, et al Heterogeneous ribonuclear protein E2 (hnRNP E2) is associated with TDP-43-immunoreactive neurites in semantic dementia but not with other TDP-43 pathological subtypes of frontotemporal lobar degeneration. Acta Neuropathol Commun. 2017;5(1):54. 28666471 10.1186/s40478-017-0454-4 PMC5493127

[23] Mori K, Lammich S, Mackenzie IRA, et al hnRNP A3 binds to GGGGCC repeats and is a constituent of p62-positive/TDP43-negative inclusions in the hippocampus of patients with C9orf72 mutations. Acta Neuropathol. 2013;125(3):413–423. 23381195 10.1007/s00401-013-1088-7

[24] Davidson YS, Flood L, Robinson AC, et al Heterogeneous ribonuclear protein A3 (hnRNP A3) is present in dipeptide repeat protein containing inclusions in frontotemporal lobar degeneration and motor neurone disease associated with expansions in C9orf72 gene. Acta Neuropathol Commun. 2017;5(1):31. 28431575 10.1186/s40478-017-0437-5 PMC5399321

[25] Bampton A, Gittings LM, Fratta P, Lashley T, Gatt A. The role of hnRNPs in frontotemporal dementia and amyotrophic lateral sclerosis. Acta Neuropathol. 2020;140(5):599–623. 32748079 10.1007/s00401-020-02203-0 PMC7547044

[26] Sidhu R, Gatt A, Fratta P, Lashley T, Bampton A. HnRNP K mislocalisation in neurons of the dentate nucleus is a novel neuropathological feature of neurodegenerative disease and ageing. Neuropathol Appl Neurobiol. 2022;48(4):e12793. 35064577 10.1111/nan.12793 PMC9208575

[27] Mackenzie IR, Neumann M. Reappraisal of TDP-43 pathology in FTLD-U subtypes. Acta Neuropathol. 2017;134(1):79–96. 28466142 10.1007/s00401-017-1716-8

[28] Hao Y, Hao S, Andersen-Nissen E, et al Integrated analysis of multimodal single-cell data. Cell. 2021;184(13):3573–3587. 34062119 10.1016/j.cell.2021.04.048 PMC8238499

[29] Lun ATL, McCarthy DJ, Marioni JC. A step-by-step workflow for low-level analysis of single-cell RNA-seq data with Bioconductor. [version 2; peer review: 3 approved, 2 approved with reservations]. F1000Res. 2016;5:2122. 27909575 10.12688/f1000research.9501.1 PMC5112579

[30] Hafemeister C, Satija R. Normalization and variance stabilization of single-cell RNA-Seq data using regularized negative binomial regression. Genome Biol. 2019;20(1):296. 31870423 10.1186/s13059-019-1874-1 PMC6927181

[31] Gittings LM, Alsop EB, Antone J, et al Cryptic exon detection and transcriptomic changes revealed in single-nuclei RNA sequencing of C9ORF72 patients spanning the ALS-FTD spectrum. Acta Neuropathol. 2023;146(3):433–450. 37466726 10.1007/s00401-023-02599-5 PMC10412668

[32] CZI Cell Science Program, Abdulla S, Aevermann B, et al CZ CELLxGENE discover: A single-cell data platform for scalable exploration, analysis and modeling of aggregated data. Nucleic Acids Res. 2025;53(D1):D886–D900. 39607691 10.1093/nar/gkae1142 PMC11701654

[33] Zhao W, Zhang S, Zhu Y, et al POSTAR3: An updated platform for exploring post-transcriptional regulation coordinated by RNA-binding proteins. Nucleic Acids Res. 2022;50(D1):D287–D294. 34403477 10.1093/nar/gkab702 PMC8728292

[34] Morabito S, Reese F, Rahimzadeh N, Miyoshi E, Swarup V. hdWGCNA identifies co-expression networks in high-dimensional transcriptomics data. Cell Rep Methods. 2023;3(6):100498. 37426759 10.1016/j.crmeth.2023.100498 PMC10326379

[35] Wu T, Hu E, Xu S, et al clusterProfiler 4.0: A universal enrichment tool for interpreting omics data. Innovation (Camb). 2021;2(3):100141. 34557778 10.1016/j.xinn.2021.100141 PMC8454663

[36] Lashley T, Rohrer JD, Bandopadhyay R, et al A comparative clinical, pathological, biochemical and genetic study of fused in sarcoma proteinopathies. Brain. 2011;134(Pt 9):2548–2564. 21752791 10.1093/brain/awr160 PMC3170529

[37] Lashley T, Holton JL, Gray E, et al Cortical alpha-synuclein load is associated with amyloid-beta plaque burden in a subset of Parkinson’s disease patients. Acta Neuropathol. 2008;115(4):417–425. 18185940 10.1007/s00401-007-0336-0

[38] Braak H, Braak E. Neuropathological stageing of Alzheimer-related changes. Acta Neuropathol. 1991;82(4):239–259. 1759558 10.1007/BF00308809

[39] Ellis RJ, Olichney JM, Thal LJ, et al Cerebral amyloid angiopathy in the brains of patients with Alzheimer’s disease: The CERAD experience, Part XV. Neurology. 1996;46(6):1592–1596. 8649554 10.1212/wnl.46.6.1592

[40] Skrobot OA, Attems J, Esiri M, et al Vascular cognitive impairment neuropathology guidelines (VCING): The contribution of cerebrovascular pathology to cognitive impairment. Brain. 2016;139(11):2957–2969. 27591113 10.1093/brain/aww214

[41] Thal DR, Rüb U, Orantes M, Braak H. Phases of A beta-deposition in the human brain and its relevance for the development of AD. Neurology. 2002;58(12):1791–1800. 12084879 10.1212/wnl.58.12.1791

[42] Montine TJ, Phelps CH, Beach TG, et al National Institute on Aging-Alzheimer’s Association guidelines for the neuropathologic assessment of Alzheimer’s disease: A practical approach. Acta Neuropathol. 2012;123(1):1–11. 22101365 10.1007/s00401-011-0910-3 PMC3268003

[43] Bankhead P, Loughrey MB, Fernández JA, et al Qupath: Open source software for digital pathology image analysis. Sci Rep. 2017;7(1):16878. 29203879 10.1038/s41598-017-17204-5 PMC5715110

[44] Gittings LM, Foti SC, Benson BC, Gami-Patel P, Isaacs AM, Lashley T. Heterogeneous nuclear ribonucleoproteins R and Q accumulate in pathological inclusions in FTLD-FUS. Acta Neuropathol Commun. 2019;7(1):18. 30755280 10.1186/s40478-019-0673-y PMC6371513

[45] Gami-Patel P, Bandopadhyay R, Brelstaff J, Revesz T, Lashley T. The presence of heterogeneous nuclear ribonucleoproteins in frontotemporal lobar degeneration with FUS-positive inclusions. Neurobiol Aging. 2016;46:192–203. 27500866 10.1016/j.neurobiolaging.2016.07.004

[46] Mikolaskova B, Jurcik M, Cipakova I, Kretova M, Chovanec M, Cipak L. Maintenance of genome stability: The unifying role of interconnections between the DNA damage response and RNA-processing pathways. Curr Genet. 2018;64(5):971–983. 29497809 10.1007/s00294-018-0819-7

[47] Naro C, Bielli P, Pagliarini V, Sette C. The interplay between DNA damage response and RNA processing: The unexpected role of splicing factors as gatekeepers of genome stability. Front Genet. 2015;6:142. 25926848 10.3389/fgene.2015.00142 PMC4397863

[48] Zhang J, Li D, Shen A, et al Expression of RBMX after spinal cord injury in rats. J Mol Neurosci. 2013;49(2):417–429. 23180094 10.1007/s12031-012-9914-2

[49] Appocher C, Mohagheghi F, Cappelli S, et al Major hnRNP proteins act as general TDP-43 functional modifiers both in Drosophila and human neuronal cells. Nucleic Acids Res. 2017;45(13):8026–8045. 28575377 10.1093/nar/gkx477 PMC5570092

[50] Bakkar N, Kovalik T, Lorenzini I, et al Artificial intelligence in neurodegenerative disease research: Use of IBM Watson to identify additional RNA-binding proteins altered in amyotrophic lateral sclerosis. Acta Neuropathol. 2018;135(2):227–247. 29134320 10.1007/s00401-017-1785-8 PMC5773659

[51] Prater KE, Latimer CS, Jayadev S. Glial TDP-43 and TDP-43 induced glial pathology, focus on neurodegenerative proteinopathy syndromes. Glia. 2022;70(2):239–255. 34558120 10.1002/glia.24096 PMC8722378

[52] Kishor A, Ge Z, Hogg JR. hnRNP L-dependent protection of normal mRNAs from NMD subverts quality control in B cell lymphoma. EMBO J. 2019;38(3):EMBJ201899128. 10.15252/embj.201899128 PMC6356069 30530525

[53] Wilkinson MF . Genetic paradox explained by nonsense. Nature. 2019;568(7751):179–180. 30962551 10.1038/d41586-019-00823-5 PMC10984164

[54] Koike Y, Pickles S, Estades Ayuso V, et al TDP-43 and other hnRNPs regulate cryptic exon inclusion of a key ALS/FTD risk gene, UNC13A. PLoS Biol. 2023;21(3):e3002028. 36930682 10.1371/journal.pbio.3002028 PMC10057836

[55] Prpar Mihevc S, Baralle M, Buratti E, Rogelj B. TDP-43 aggregation mirrors TDP-43 knockdown, affecting the expression levels of a common set of proteins. Sci Rep. 2016;6:33996. 27665936 10.1038/srep33996 PMC5036055

[56] Arseni D, Chen R, Murzin AG, et al TDP-43 forms amyloid filaments with a distinct fold in type A FTLD-TDP. Nature. 2023;620(7975):898–903. 37532939 10.1038/s41586-023-06405-w PMC10447236

[57] Arseni D, Hasegawa M, Murzin AG, et al Structure of pathological TDP-43 filaments from ALS with FTLD. Nature. 2022;601(7891):139–143. 34880495 10.1038/s41586-021-04199-3 PMC7612255

[58] Arseni D, Nonaka T, Jacobsen MH, et al Heteromeric amyloid filaments of ANXA11 and TDP-43 in FTLD-TDP type C. Nature. 2024;634(8034):662–668. 39260416 10.1038/s41586-024-08024-5 PMC11485244

[59] Laferrière F, Maniecka Z, Pérez-Berlanga M, et al TDP-43 extracted from frontotemporal lobar degeneration subject brains displays distinct aggregate assemblies and neurotoxic effects reflecting disease progression rates. Nat Neurosci. 2019;22(1):65–77. 30559480 10.1038/s41593-018-0294-y

[60] Dormann D, Rodde R, Edbauer D, et al ALS-associated fused in sarcoma (FUS) mutations disrupt transportin-mediated nuclear import. EMBO J. 2010;29(16):2841–2857. 20606625 10.1038/emboj.2010.143 PMC2924641

[61] Wek RC . Role of eIF2α kinases in translational control and adaptation to cellular stress. Cold Spring Harb Perspect Biol. 2018;10(7):a032870. 29440070 10.1101/cshperspect.a032870 PMC6028073

[62] Sonobe Y, Aburas J, Krishnan G, et al A C. elegans model of C9orf72-associated ALS/FTD uncovers a conserved role for eIF2D in RAN translation. Nat Commun. 2021;12(1):6025. 34654821 10.1038/s41467-021-26303-x PMC8519953

[63] Broce IJ, Sirkis DW, Nillo RM, et al C9orf72 gene networks in the human brain correlate with cortical thickness in C9-FTD and implicate vulnerable cell types. *Front Neurosci*. 2024;18:1258996. [eCollection 2024]. doi: 10.3389/fnins.2024.1258996 PMC10925697 38469573

[64] Maity S, Fiore APZP, An D, et al Molecular signatures of proteostasis control in motor neurons with different sensitivity to Amyotrophic Lateral Sclerosis. BioRxiv. [Preprint]. doi:10.1101/2022.04.10.487765

[65] Mehta AR, Gregory JM, Dando O, et al Mitochondrial bioenergetic deficits in C9orf72 amyotrophic lateral sclerosis motor neurons cause dysfunctional axonal homeostasis. Acta Neuropathol. 2021;141(2):257–279. 33398403 10.1007/s00401-020-02252-5 PMC7847443

[66] Nickerson KR, Sammoura FM, Zhou Y, Jaworski A. Slit-robo signaling supports motor neuron avoidance of the spinal cord midline through DCC antagonism and other mechanisms. Front Cell Dev Biol. 2025;13:1563403. 40276653 10.3389/fcell.2025.1563403 PMC12018395

[67] Li J, Jaiswal MK, Chien J-F, et al Divergent single cell transcriptome and epigenome alterations in ALS and FTD patients with C9orf72 mutation. Nat Commun. 2023;14(1):5714. 37714849 10.1038/s41467-023-41033-y PMC10504300

[68] Marsan E, Velmeshev D, Ramsey A, et al Astroglial toxicity promotes synaptic degeneration in the thalamocortical circuit in frontotemporal dementia with GRN mutations. J Clin Invest. 2023;133(6):e164919. 36602862 10.1172/JCI164919 PMC10014110

[69] Ling S-C, Polymenidou M, Cleveland DW. Converging mechanisms in ALS and FTD: Disrupted RNA and protein homeostasis. Neuron. 2013;79(3):416–438. 23931993 10.1016/j.neuron.2013.07.033 PMC4411085

[70] Fodder K, Murthy M, Rizzu P, et al Brain DNA methylomic analysis of frontotemporal lobar degeneration reveals OTUD4 in shared dysregulated signatures across pathological subtypes. Acta Neuropathol. 2023;146(1):77–95. 37149835 10.1007/s00401-023-02583-z PMC10261190

[71] Doukhanine E, Gavino C, Haines JD, Almazan G, Richard S. The QKI-6 RNA binding protein regulates actin-interacting protein-1 mRNA stability during oligodendrocyte differentiation. Mol Biol Cell. 2010;21(17):3029–3040. 20631256 10.1091/mbc.E10-04-0305 PMC2929996

[72] Larocque D, Galarneau A, Liu H-N, Scott M, Almazan G, Richard S. Protection of p27(Kip1) mRNA by quaking RNA binding proteins promotes oligodendrocyte differentiation. Nat Neurosci. 2005;8(1):27–33. 15568022 10.1038/nn1359

[73] Li Z, Zhang Y, Li D, Feng Y. Destabilization and mislocalization of myelin basic protein mRNAs in quaking dysmyelination lacking the QKI RNA-binding proteins. J Neurosci. 2000;20(13):4944–4953. 10864952 10.1523/JNEUROSCI.20-13-04944.2000 PMC6772302

[74] Zhao L, Ku L, Chen Y, Xia M, LoPresti P, Feng Y. QKI binds MAP1B mRNA and enhances MAP1B expression during oligodendrocyte development. Mol Biol Cell. 2006;17(10):4179–4186. 16855020 10.1091/mbc.E06-04-0355 PMC1635361

[75] García-Ruiz S, Zhang D, Gustavsson EK, et al Splicing accuracy varies across human introns, tissues, age and disease. Nat Commun. 2025;16(1):1068. 39870615 10.1038/s41467-024-55607-x PMC11772838

[76] Rhine K, Li R, Kopalle HM, et al Neuronal aging causes mislocalization of splicing proteins and unchecked cellular stress. Nat Neurosci. 2025;28(6):1174–1184. 40456907 10.1038/s41593-025-01952-z PMC12148940

[77] Trautwig AN, Shantaraman A, Chung M, et al Molecular subtyping based on hippocampal cryptic exon burden reveals proteome-wide changes associated with TDP-43 pathology across the spectrum of LATE and Alzheimer’s Disease. bioRxiv. [Preprint]. doi:10.1101/2025.05.30.656396 41860868

[78] Neumann M, Kwong LK, Truax AC, et al TDP-43-positive white matter pathology in frontotemporal lobar degeneration with ubiquitin-positive inclusions. J Neuropathol Exp Neurol. 2007;66(3):177–183. 17356379 10.1097/01.jnen.0000248554.45456.58

[79] Heo D, Ling JP, Molina-Castro GC, et al Stage-specific control of oligodendrocyte survival and morphogenesis by TDP-43. eLife. 2022;11:e75230. 35311646 10.7554/eLife.75230 PMC8970587

[80] Wang J, Ho WY, Lim K, et al Cell-autonomous requirement of TDP-43, an ALS/FTD signature protein, for oligodendrocyte survival and myelination. Proc Natl Acad Sci U S A. 2018;115(46):E10941–E10950. 30373824 10.1073/pnas.1809821115 PMC6243235

[81] Wang P, Deng J, Dong J, et al TDP-43 induces mitochondrial damage and activates the mitochondrial unfolded protein response. PLoS Genet. 2019;15(5):e1007947. 31100073 10.1371/journal.pgen.1007947 PMC6524796

[82] Moore KM, Nicholas J, Grossman M, et al Age at symptom onset and death and disease duration in genetic frontotemporal dementia: An international retrospective cohort study. Lancet Neurol. 2020;19(2):145–156. 31810826 10.1016/S1474-4422(19)30394-1 PMC7007771

