# Supplementary material for: Transcriptomic and pathological analysis of the hnRNP network reveals glial involvement in frontotemporal lobar degeneration pathological subtypes
Source: Brain Commun. 2026 Jun 1;8(3):fcag197. doi: 10.1093/braincomms/fcag197 (PMC13276875; doi:10.1093/braincomms/fcag197)
Supplement: fcag197_Supplementary_Data [file fcag197_supplementary_data.zip › Supplementary_Tables_1-3.pdf]

Supplementary Table 1

Case no	Path Diag	Mutations	Clinical diagnosis	AAO (y)	AAD (y)	Disease duration (y)	Sex	% TDP43 affected neurons Hippocampus	Frontal cortex inclusions	Frontal cortex neurites	Frontal cortex pathology (combined)	Temporal cortex inclusions	Temporal cortex neurites	Temporal cortex pathology (combined)	Included in HnRNP immunohistochemical study	Included in ScRNASeq analysis
1	TDP A		bvFTD	57	62	5	M	2.6	0.8	1.1	1.9	1.4	5.3	6.7	✓	
2	TDP A		CBD	51	61	10	M	0.7	1.8	24.0	25.8	2.7	20.2	22.9		✓
3	TDP A		PNFA	66	72	6	M	0.2	0.8	0.8	1.5	0.5	4.1	4.6		✓
4	TDP A		Control	77	79	2	M	0.9	0.3	0.4	0.7	0.3	10.8	11.1	✓	
5	TDP A		FTD	70	83	13	F	0.6	2.9	0.4	3.4	1.6	0.3	1.9	✓	
6	TDP A		FTD-MND	75	79	4	F	5.8	0.8	5.5	6.3	0.1	0.2	0.3	✓	
7	TDP A		MND	47	53	6	M	1.1	0.1	0.6	0.7	0.6	0.0	0.6	✓	✓
8	TDP A		PSP	83	87	4	F	3.4	0.1	0.5	0.6	1.8	2.5	4.4	✓	
18	TDP A	*C9orf72*	PNFA	56	67	11	F	18.6	0.4	0.1	0.5	1.6	4.6	6.2	✓	
19	TDP A	*C9orf72*	Picks	54	60	6	M	3.0	0.7	0.1	0.8				✓	
20	TDP A	*C9orf72*	PNFA	57	62	5	F	6.3	1.1	0.4	1.5	1.3	0.2	1.4	✓	✓
21	TDP A	*C9orf72*	Picks	53	63	10	M	31.6	0.2	0.0	0.2	2.0	5.6	7.6	✓	✓
22	TDP A	*C9orf72*	FTD-MND	66	74	8	F	2.4	0.0	0.6	0.6	0.2	0.2	0.4	✓	
23	TDP A	*C9orf72*	Picks	62	68	6	M	16.5	0.0	0.6	0.6	0.0	0.0	0.0	✓	
24	TDP A	*C9orf72*	bvFTD	43	45	2	M	1.9	0.1	0.0	0.1	0.6	0.0	0.6	✓	
25	TDP A	*C9orf72*	FTD-MND	66	71	5	M	4.5	1.6	0.4	2.0	0.1	0.0	0.1		✓
26	TDP A	*C9orf72*	bvFTD	58	66	8	F	8.0	0.2	0.0	0.2	0.9	0.0	0.9		
27	TDP C		PNFA	77	80	3	F	2.7	0.9	3.4	4.3	1.8	2.6	4.4		
28	TDP C		SD	64	78	14	M	6.6	0.0	0.2	0.2	6.9	4.3	11.1	✓	✓
29	TDP C		SD	58	72	14	F	13.1	0.0	15.9	15.9	0.0	12.5	12.5		✓
30	TDP C		SD	66	76	10	M	11.3	0.0	9.6	9.7	0.1	9.7	9.8		✓*
31	TDP C		SD	50	65	15	M	12.6							✓	
32	TDP C		FTD	61	66	5	M	50.9	0.0	1.3	1.3	0.2	4.8	5.1	✓	
33	TDP C		SD	58	73	15	F	5.0	0.2	11.0	11.2	0.0	0.4	0.4	✓	✓
34	TDP C		SD	64	74	10	M	30.3	0.0	2.6	2.6	0.0	3.1	3.1	✓	✓*
35	TDP C		SD	59	73	14	F	4.1	0.1	7.0	7.1	0.7	6.2	6.8	✓	✓
36	TDP C		SD	44	67	23	M	46.8	0.4	18.5	18.9	0.5	4.4	4.9	✓	
37	TDP C		PSP	60	65	5	M	0.5				0.1	0.1	0.2	✓	
38	Control			n/a	79	n/a	F	0	0	0	0	0	0	0	✓	
39	Control			n/a	69	n/a	M	0	0	0	0	0	0	0	✓	✓
40	Control			n/a	38	n/a	M	0	0	0	0	0	0	0	✓	
41	Control			n/a	94	n/a	F	0	0	0	0	0	0	0	✓	
42	Control			n/a	85	n/a	M	0	0	0	0	0	0	0	✓	
43	Control			n/a	92	n/a	F	0	0	0	0	0	0	0	✓	
44	Control			n/a	71	n/a	F	0	0	0	0	0	0	0		✓
45	Control			n/a	86	n/a	F	0	0	0	0	0	0	0		
46	Control			n/a	68	n/a	F	0	0	0	0	0	0	0		
47	Control			n/a	80	n/a	F	0	0	0	0	0	0	0		✓
48	Control			n/a	93	n/a	F	0	0	0	0	0	0	0		
49	Control			n/a	83	n/a	F	0	0	0	0	0	0	0		✓
50	Control			n/a	71	n/a	M	0	0	0	0	0	0	0		
51	Control			n/a	101	n/a	M	0	0	0	0	0	0	0		✓

Key: 0 10 20 30 40 50 60

Supplementary Table 2

Antibody	Antibody Source	Cat.No	Specificity	Dilution	Antigen
TDP-43	Cosmo	TIP-PTD-P02	Rb	1:10,000	pS409/410
hnRNP A1	Abcam	ab5832	Ms	1:100	Full Length
hnRNP A2B1	Abcam	ab6102	Ms	1:100	-
hnRNP C1/C2	Abcam	ab97541	Rb	1:100	aa 1-152
hnRNPD1/D2	abcam	ab61193	Rb	1:500	Phospho serine 83
hnRNPE1/E2	Santa Cruz	sc-28725	Rb	1:100	aa 171-280
hnRNP F	abcam	ab50982	Rb	1:100	aa 361-410
hnRNP G	abcam	ab70064	Rb	1:200	N-terminus
hnRNP H	abcam	ab10374	Rb	1:200	aa 400-448
hnRNP I	Santa Cruz	sc-16549	Gt	1:100	internal sequence
hnRNP L	abcam	ab6106	Ms	1:1000	Full length
hnRNP M	Sigma	HPA024344	Rb	1:1000	aa 344-448
hnRNP P (FUS)	Novus	NB100-565	Rb	1:200	aa 1-50
hnRNP Q	Thermo	PA5-15009	Rb	1:200	aa 591-623
hnRNP R	Abcam	ab30930	Rb	1:200	-
hnRNP U	abcam	ab10297	Ms	1:1000	Full length

Supplementary Table 3

HnRNP A1

	Inclusions	Nucleus	Cytoplasm
TDP A	0 (0-0)	2 (1-2)	1 (1-2)
TDP A-C9	0 (0-0)	2 (2-3)	2 (1-2)
TDP C	0 (0-0)	2 (2-3)	2 (1-2)
Controls	0 (0-0)	3 (3-3)	2 (2-2)
	H=0	H=13.791	H=9.913
	p=1.00	**p=0.003**	**p=0.019**

HnRNP A2B1

	Inclusions	Nucleus	Cytoplasm
TDP A	0 (0-0)	3 (3-3)	1 (1-2)
TDP A-C9	0 (0-0)	3 (2-3)	2 (1-2)
TDP C	0 (0-0)	3 (2-3)	2 (1-2)
Controls	0 (0-0)	3 (3-3)	0 (0-1)
	H=0	H=3.468	H=17.501
	p=1.00	p=0.325	**p=<0.001**

HnRNP C

	Inclusions	Nucleus	Cytoplasm
TDP A	0 (0-0)	3 (3-3)	0 (0-0)
TDP A-C9	0 (0-0)	3 (3-3)	0 (0-0)
TDP C	0 (0-0)	3 (3-3)	0 (0-0)
Controls	0 (0-0)	3 (3-3)	1 (0-1)
	H=0	H=0	H=15.826
	p=1.00	p=1.00	**p=0.001**

HnRNP D

	Inclusions	Nucleus	Cytoplasm
TDP A	0 (0-0)	2 (2-3)	0 (0-1)
TDP A-C9	0 (0-0)	2 (2-3)	1 (0-1)
TDP C	0 (0-0)	3 (3-3)	2 (1-2)
Controls	0 (0-0)	3 (3-3)	0 (0-0)
	H=0	H=11.243	H=18.614
	p=1.00	**p=0.01**	**p=<0.001**

HnRNP E1/2

	Inclusions	Nucleus	Cytoplasm
TDP A	0 (0-0)	0 (0-0)	0 (0-2)
TDP A-C9	0 (0-0)	0 (0-0)	0 (0-1)
TDP C	1 (0-1)	2 (1-2)	1 (1-2)
Controls	0(0-0)	0(0-0)	1 (1-1)
	H=16.156	H=24.504	H=9.096
	**p=0.001**	**p=<0.001**	**p=0.028**

HnRNP F

	Inclusions	Nucleus	Cytoplasm
TDP A	0 (0-0)	0 (0-1)	3 (3-3)
TDP A-C9	0 (0-0)	1 (0-1)	3 (3-3)
TDP C	0 (0-0)	0 (0-1)	2.5 (2-3)
Controls	0 (0-0)	0 (0-0)	3 (3-3)
	H=0	H=5.574	H=10.739
	p=1.00	p=0.134	**p=0.013**

HnRNP G

	Inclusions	Nucleus	Cytoplasm
TDP A	1 (1-1)	1 (1-1)	1 (1-1)
TDP A-C9	1 (1-1)	1 (1-2)	1 (1-1)
TDP C	1 (0-1)	1 (1-1)	1 (1-2)
Controls	0 (0-0)	1 (1-1)	2 (2-2)
	H=18.552	H=2.714	H=15.097
	**p=<0.001**	p=0.438	**p=0.002**

HnRNP H

	Inclusions	Nucleus	Cytoplasm
TDP A	0 (0-0)	2 (2-3)	0.5 (0-2)
TDP A-C9	0 (0-0)	2 (2-3)	1.5 (1-2)
TDP C	0 (0-0)	3 (2-3)	2 (2-3)
Controls	0 (0-0)	3 (3-3)	0 (0-0)
	H=0	H=13.004	H=17.056
	p=1.00	**p=0.005**	**p=<0.001**

HnRNP I

	Inclusions	Nucleus	Cytoplasm
TDP A	0 (0-0)	2 (2-2)	1 (0-2)
TDP A-C9	0 (0-0)	2 (2-2)	1 (0-1)
TDP C	0 (0-0)	2 (2-3)	1 (1-2)
Controls	0 (0-0)	3 (3-3)	0.5 (0-1)
	H=0	H=17.717	H=9.673
	p=1.00	**p=<0.001**	p=0.22

HnRNP L

	Inclusions	Nucleus	Cytoplasm
TDP A	0 (0-0)	3 (3-3)	1 (0-1)
TDP A-C9	0 (0-0)	3 (3-3)	1 (0-3)
TDP C	0 (0-0)	3 (3-3)	2 (1-3)
Controls	0 (0-0)	3 (3-3)	2 (1-2)
	H=0	H=0	H=7.786
	p=1.00	p=1.00	P=0.051

HnRNP M

	Inclusions	Nucleus	Cytoplasm
TDP A	0 (0-0)	3 (3-3)	0 (0-0)
TDP A-C9	0 (0-0)	3 (3-3)	0 (0-0)
TDP C	0 (0-0)	3 (3-3)	0 (0-0)
Controls	0 (0-0)	3 (3-3)	0 (0-0)
	H=0	H=0	H=0
	p=1.00	p=1.00	p=1.00

HnRNP P (FUS)

	Inclusions	Nucleus	Cytoplasm
TDP A	0 (0-0)	3 (2-3)	0 (0-0)
TDP A-C9	0 (0-0)	3 (3-3)	0 (0-1)
TDP C	0 (0-0)	3 (3-3)	1 (1-2)
Controls	0 (0-0)	3 (3-3)	2 (1-2)
	H=0	H=4.000	H=19.284
	p=1.00	p=0.261	**p=<0.001**

HnRNP Q

	Inclusions	Nucleus	Cytoplasm
TDP A	0.5 (0-1)	1.5 (1-3)	1 (1-2)
TDP A-C9	1 (0-1)	2 (1-3)	1 (1-2)
TDP C	1 (0-1)	3 (2-3)	2 (1-2)
Controls	0 (0-0)	2.5 (2-3)	1.5 (1-2)
	H=5.584	H=10.422	H=7.405
	p=0.134	**p=0.015**	p=0.06

HnRNP R

	Inclusions	Nucleus	Cytoplasm
TDP A	0 (0-1)	2.5 (0-3)	2 (0-3)
TDP A-C9	0 (0-1)	2 (0 -3)	1.5 (0-3)
TDP C	0 (0-1)	2 (2-3)	2 (2-3)
Controls	0 (0-0)	2.5 (1-3)	2 (1-2)
	H=1.962	H=0.807	H=1.629
	p=0.580	p=0.848	p=0.653

HnRNP U

TDP A	Inclusions	Nucleus	Cytoplasm
TDP A-C9	0 (0-0)	2.5 (1-3)	0 (0-0)
TDP C	0 (0-0)	2 (2-3)	0 (0-0)
Controls	0 (0-0)	2 (2-3)	0 (0-0)
	0 (0-0)	2 (2-2)	0 (0-0)
	H=0	H=2.147	H=0
	p=1.00	p=0.542	p=1.00
