# Supplementary material for: Transcriptomic and pathological analysis of the hnRNP network reveals glial involvement in frontotemporal lobar degeneration pathological subtypes
Source: Brain Commun. 2026 Jun 1;8(3):fcag197. doi: 10.1093/braincomms/fcag197 (PMC13276875; doi:10.1093/braincomms/fcag197)
Supplement: fcag197_Supplementary_Data [file fcag197_supplementary_data.zip › Supplementary_table_and_figure_legends.docx]

**Supplementary tables and figures:**

**Supplementary Table 1:** Full demographic data of all the cases used in the study, including a heatmap of variable phospho-TDP-43 pathology. AAO = age at onset. AAD = age at death. y = years. In the clinical diagnosis column: bvFTD=behavioural variant FTD, CBD = corticobasal degeneration, PNFA = progressive non-fluent aphasia, MND = motor neuron disease, FTD = frontotemporal dementia, PSP = progressive supranuclear palsy, Picks = Picks disease, SD = semantic dementia. In the pathology heatmap, a red gradient in the cells indicates higher levels of pathology. These numbers correspond to the ones graphically represented in Figure 1. In the TDP C group, an asterisk (*) next to the tick denotes the two cases which were later eliminated from scRNAseq analysis after not passing quality checks.

**Supplementary Table 2: List of antibodies used in the immunohistochemical study.** List of the antibody source, specificity, dilution and the antigen the antibody recognised.

**Supplementary Table 3:** **Semi-quantification of the immunohistochemical analysis of the hnRNPs in different FTLD subtypes.** Values are listed in the range 0-3 where 0 = staining is absent, 1 = few cells stained, 2= moderate number of cells stained and 3 = many cells stained. Differences in group distribution (TDP A (n=6), TDP A-*C9* (n=7),TDP C (n=8) and neurologically normal controls (n=6)) are statistically analysed via a Kruskal-Wallis K independent sample test. H indicates the H-test statistic and the p value indicates statistical significance. Statistically significant tests where p<0.05 are written in bold, suggesting there is a significant altered distribution of the HnRNP in an FTLD subtype compared to controls.

**Supplementary Table 4:** Summary table of the number of nuclei collected for single-nuclei RNA sequencing across each case and how they distribute across different clusters with differential gene expression results across all cell types. The workflow followed is outlined in Figure 3.

**Supplementary Table 5:** Summary table of the canonical pathways enriched across cell types in the single-nuclei RNA sequencing dataset.

**Supplementary Table 6:** Module eigengene based connectivity across modules in the FTLD-TDP snRNAseq dataset

**Supplementary Figure 1:** Demographics comparison of FTLD subtypes (TDP A; n=8, TDP A-*C9*; n=9, and TDP C; n=11) against TDP-43 pathological scoring in the (A) temporal cortex, (B) frontal cortex, and (C) hippocampal granule cell layer. Dotplots contain the comparison of FTLD subtypes for (D) age of onset in years, (E) age at death in years, (F) disease duration in years, and (G) *post-mortem* interval in hours. N=11 for controls in E and G. Data were analysed using a simple linear regression analysis and Kruskal Wallis analysis, respectively. In A-G data points represent individual cases. ** indicates p<0.01.

**Supplementary Figure 2:** HnRNP immunohistochemistry and quantification in different FTLD subtypes (TDP A; n=6, TDP A-*C9*; n=7,TDP C; n=8) and neurologically normal controls (n=6). Immunohistochemical images of HnRNP A1, A2B1, C1/2, D and E1/2 staining in the frontal cortex in all FTLD subtypes as well as controls. The violin plots next to the corresponding HnRNP images depict the values listed in Supplementary Table 3 where score ranges from 0 to 3 :0 = staining is absent, 1 = few cells stained, 2= moderate number of cells stained and 3 = many cells stained. Scale bar indicates 50µm. In all plots N=Nuclear, C= Cytoplasmic, I= inclusions.

**Supplementary Figure 3:** HnRNP immunohistochemistry and quantification in different FTLD subtypes (TDP A; n=6, TDP A-*C9*; n=7, TDP C; n=8) and neurologically normal controls (n=6). Immunohistochemical images of HnRNP F, G, H, I, and L staining in the frontal cortex in all FTLD subtypes as well as controls. The violin plots next to the corresponding HnRNP images depict the values listed in Supplementary Table 3 where score ranges from 0 to 3 :0 = staining is absent, 1 = few cells stained, 2= moderate number of cells stained and 3 = many cells stained. Scale bar indicates 50µm. In all plots N=Nuclear, C= Cytoplasmic, I= inclusions.

**Supplementary Figure 4:** HnRNP immunohistochemistry and quantification in different FTLD subtypes (TDP A; n=6, TDP A-*C9*; n=7,TDP C; n=8) and neurologically normal controls (n=6). Immunohistochemical images of HnRNP M, P, Q, R and U staining in the frontal cortex in all FTLD subtypes as well as controls. The violin plots next to the corresponding HnRNP images depict the values listed in Supplementary Table 3 where score ranges from 0 to 3 :0 = staining is absent, 1 = few cells stained, 2= moderate number of cells stained and 3 = many cells stained. Scale bar indicates 50µm. In all plots N=Nuclear, C= Cytoplasmic, I= inclusions.

**Supplementary Figure 5:** Most significant differentially expressed genes in different cell types across different FTLD subtypes: Heatmaps displaying log₂ fold change of the top 75 most significantly differentially expressed genes in L2-3 and L3-5 excitatory neurons, across FTLD subtypes (TDP A; n=3, TDP A-*C9*; n=3, and TDP C; n=4) compared to controls (n=5), calculated using a Wilcoxon rank-sum test and applies a Bonferroni correction across all tested genes to calculate adjusted p-values.

**Supplementary Figure 6:** SnRNA-seq analysis of the genes belonging to the HnRNP network from the published datasets: Heatmaps showing the differential gene expression of TDP A-*C9* cases against controls from Gittings *et al.* (2023) and Li *et al.* (2023); syn45351388 (control; n=12 vs. FTLD-TDPA *C9*; n=9) and GSE219281 (control; n=6 vs. FTD-C9; n=5), respectively. Red- and blue-coloured boxes indicate a positive and negative log₂ fold change, respectively, with an asterisk indicating a false discovery rate of <0.05, calculated using a Wilcoxon rank-sum test and applies a Bonferroni correction across all tested genes.

.
